# Comparative genomic analysis and optimization of astaxanthin production of *Rhodotorula paludigena* TL35-5 and *Rhodotorula sampaioana* PL61-2

**DOI:** 10.1371/journal.pone.0304699

**Published:** 2024-07-12

**Authors:** Patcharaporn Hoondee, Sukanya Phuengjayaem, Engkarat Kingkaew, Pornchai Rojsitthisak, Boonchoo Sritularak, Somphob Thompho, Natapol Pornputtapong, Worathat Thitikornpong, Somboon Tanasupawat

**Affiliations:** 1 Department of Biochemistry and Microbiology, Faculty of Pharmaceutical Sciences, Chulalongkorn University, Bangkok, Thailand; 2 Division of Biology, Faculty of Science and Technology, Rajamangala University of Technology Krungthep, Bangkok, Thailand; 3 Department of Microbiology, Faculty of Science, King Mongkut’s University of Technology Thonburi, Bangkok, Thailand; 4 Department of Biology, School of Science, King Mongkut’s Institute of Technology Ladkrabang, Bangkok, Thailand; 5 Department of Food and Pharmaceutical Chemistry, Faculty of Pharmaceutical Sciences, Chulalongkorn University, Bangkok, Thailand; 6 Department of Pharmacognosy and Pharmaceutical Botany, Faculty of Pharmaceutical Sciences, Chulalongkorn University, Bangkok, Thailand; 7 Pharmaceutical Research Instrument Center, Faculty of Pharmaceutical Sciences, Chulalongkorn University, Bangkok, Thailand; Zhengzhou University, CHINA

## Abstract

Astaxanthin is a powerful antioxidant known to enhance skin, cardiovascular, eye, and brain health. In this study, the genome insights and astaxanthin production of two newly isolated astaxanthin-producing yeasts (TL35-5 and PL61-2) were evaluated and compared. Based on their phenotypic and genotypic characteristics, TL35-5 and PL61-2 were identified as basidiomycetous yeasts belonging to *Rhodotorula paludigena* and *Rhodotorula sampaioana*, respectively. To optimize astaxanthin production, the effects of cultural medium composition and cultivation conditions were examined. The optimal conditions for astaxanthin production in *R*. *paludigena* TL35-5 involved cultivation in AP medium containing 10 g/L glucose as the sole carbon source, supplemented with 1.92 g/L potassium nitrate, pH 6.5, and incubation at 20°C for 3 days with shaking at 200 rpm. For *R*. *sampaioana* PL61-2, the optimal medium composition for astaxanthin production consisted of AP medium with 40 g/L glucose, supplemented with 0.67 g/L urea, pH 7.5, and the fermentation was carried out at 20°C for 3 days with agitating at 200 rpm. Under their optimal conditions, *R*. *paludigena* TL35-5 and *R*. *sampaioana* PL61-2 gave the highest astaxanthin yields of 3.689 ± 0.031 and 4.680 ± 0.019 mg/L, respectively. The genome of TL35-5 was 20,982,417 bp in length, with a GC content of 64.20%. A total of 6,789 protein-encoding genes were predicted. Similarly, the genome of PL61-2 was 21,374,169 bp long, with a GC content of 64.88%. It contained 6,802 predicted protein-encoding genes. Furthermore, all essential genes involved in astaxanthin biosynthesis, including *CrtE*, *CrtYB*, *CrtI*, *CrtS*, and *CrtR*, were identified in both *R*. *paludigena* TL35-5 and *R*. *sampaioana* PL61-2, providing evidence for their ability to produce astaxanthin.

## Introduction

Nowadays, the global health, wellness, and nutraceuticals market is expanding as people become more concerned about their health in the aftermath of the COVID-19 pandemic [1]. Carotenoids are among the various bioactive compounds used commercially as food supplements, animal feed supplements, natural food colorants, nutritional supplements, and nutraceuticals for cosmetic and pharmaceutical purposes [2, 3].

Astaxanthin (3,3’-dihydroxy-4,4’-diketo-β-β carotene) is a red-colored carotenoid belonging to the xanthophyll class. This compound is likely the most important of the highly valued ketocarotenoids because it is a powerful antioxidant, anti-inflammatory, and anticancer agent with a significant impact on human health. It improves the immune system, prevents cardiovascular disease, various cancers, diabetes, liver disease, stomach problems, obesity, and cell aging [2, 4–7]. Due to its many biological activities, astaxanthin is widely used in various fields such as pharmaceuticals, food, cosmetics, health care, and aquaculture [5]. According to recent estimates, the global astaxanthin market is projected to grow at a 17.1% annual rate, reaching $7.28 billion by 2030, up from $2.83 billion in 2023 [8]. However, most commercially available astaxanthin and other high-value carotenoids, such as β-carotene and canthaxanthin, are primarily synthesized chemically [3]. Some research suggests that synthetic carotene is carcinogenic [9]. Moreover, natural astaxanthin derived from *Haematococcus pluvialis* has a greater effect on antioxidant enzyme levels in rats than synthetic astaxanthin [10]. This has been a major reason for exploring alternative methods of producing natural astaxanthin and other carotenoids.

Various microorganisms have been reported as natural astaxanthin producers [11]. Among them, the green alga *Haematococcus pluvialis* is the major commercial astaxanthin-producing microorganism [12]. Although *H*. *pluvialis* has high concentrations of astaxanthin, industrial applications are limited by lengthy autotrophic cultivation in open freshwater ponds and the necessity of disrupting the cell wall to liberate the astaxanthin. The pigmented yeast *Xanthophyllomyces dendrorhous* (formerly *Phaffia rhodozyma*) is a promising producer of astaxanthin [11, 13]. Yeast has advantages over microalgae because it grows quickly without being affected by changing climatic or seasonal conditions, requires less land for cultivation than plant cultivation, and is easier to scale up production than single-celled algae due to its ability to use diverse carbon sources [14]. Besides *X*. *dendrorhous*, another pigmented yeast, *Rhodosporiidium toruloides* VN1, and *Rhodotorula* sp. CP72-2, are able to produce astaxanthin naturally [15, 16]. However, little is known about the whole genome sequence of *Rhodotorula* species and its genes involved in astaxanthin biosynthesis. In addition, fermentation optimization has been extensively explored as a potent method to enhance titers, yields, and productivities. Astaxanthin production is generally influenced by various parameters of medium composition and cultural conditions. Optimization is a powerful tool that can rapidly improve titers, yields, and productivities, as well as reduce production costs, which was the goal of basic research for industrial applications. The "One-factor-at-a-time" technique is favored and widely used for fermentation optimization. This method involves changing one factor while keeping the others constant. Although simple, it frequently necessitates a significant amount of time and effort. Thus, understanding the effect of medium composition and cultural conditions on the growth and astaxanthin production is necessary.

During a study of the diversity of culturable yeast associated with flowers in Thailand, two pigmented yeast strains (TL35-5 and PL61-2) were isolated, and their astaxanthin-producing potential was investigated. As a result, they were identified as astaxanthin-producing yeast. Therefore, this study aims to identify and characterize two newly isolated astaxanthin-producing yeasts, TL35-5 and PL61-2. Additionally, we sequenced their entire genome and performed comparative genomic analysis to gain insight into the astaxanthin biosynthesis pathway of their species and, more broadly, the genus *Rhodotorula*. Furthermore, the medium composition and cultural condition parameters that could influence the growth and astaxanthin production of these specific yeasts were optimized for the first time.

## Materials and methods

### Isolation and identification of astaxanthin-producing yeasts

Two yeast isolates, TL35-5 and PL61-2, were isolated and maintained on Yeast extract malt extract (YM) agar plates with a pH of 5.5. The plates were incubated at 30°C for 72 h. For long-term storage, the cultures were preserved in the skim milk and glycerol stocks at -20 °C for further studies.

Yeast morphological and physiological characteristics were determined by the standard method [17]. Cell and colony morphology were studied using yeast grown on 5% (w/v) malt extract agar at 25 °C for 7 days. Yeast cell morphology was observed under light microscopy at ×400 magnifications, and colony morphology characteristics were visually recorded. Carbon assimilation tests were conducted using ID 32 C kit (BioMerieux, France) by following manufacture instruction. Pseudo hyphae or true hyphae production was examined on corn meal agar using the Dalmau slide culture technique and incubated at 25 °C for 21 days.

For genotypic identification, genomic DNA was extracted from three-day-old yeast using a modified version of the method reported by Pranimit et al. [18]. The LSU D1/D2 domain (26S rRNA gene) was amplified through polymerase chain reaction (PCR) using the NL1 (5’-GCATATCAATAAGCGGAGGAAAAG-3’) and NL4 (5’-GGTCCGTGTTTCAAGACGG-3’) primers under previously described conditions [19]. The PCR product obtained was purified using the Gel/PCR DNA fragment extraction kit (Geneaid Biotech Ltd., Taiwan). Purified PCR products were sequenced bidirectionally using the BT sequencing technique (Celemics, Inc., Republic of Korea). The resulting sequences were manually edited using the BioEdit program and compared to those available in the online database of the NCBI GenBank using the BLASTn program. The sequences of the isolates and related strains were aligned using the MUSCLE program [20]. A phylogenetic tree was constructed using MEGA 11, employing the neighbor-joining (NJ) method with the Kimura two-parameter model and 1000 bootstrap replications [21–23]. The nucleotide sequences were deposited in the NCBI GenBank database.

### Optimization of astaxanthin production

#### Inoculum preparation and astaxanthin production procedure

The one loopful of the two-day-old yeast grown on YM agar at 25 °C was inoculated into YM broth (50 mL) in a 250-mL Erlenmeyer flask and incubated at 30 °C and 200 rpm for 48 h [24]. The resultant culture was collected by centrifugation (6,500 × g, 10 min). The obtained cells were resuspended in 0.85% normal saline until the OD660 reached 1.00 ± 0.1. Then, 10% (v/v) of inoculum were transferred into 50 ml of astaxanthin production (AP) medium (10 g/L of glucose, 3 g/L of yeast extract, 3 g/L of malt extract, and 5 g/L of peptone, pH 5.5) in a 250-mL Erlenmeyer flask, and incubated at 25 °C and 200 rpm for 3 d. Cells were collected by centrifugation (4 °C, 7,745 × g, 10 min) and were then washed twice with sterile distilled water. The obtained cells were dried by lyophilization and weighed to obtain the dry cell weight (DCW). The lyophilized cells were subjected to further analysis.

*Astaxanthin production optimization*. The optimization of astaxanthin production of *R*. *paludigena* TL35-5 and *R*. *sampaioana* PL61-2 in the AP medium was investigated using the one factor at a time method by: 1) varying the type of carbon source, including 10 g/L of glucose, 10 g/L of sucrose, 10 g/L of fructose, or 10 g/L of maltose; 2) varying concentration of the selected carbon source (10, 20, 30, 40, or 50 g/L); 3) varying nitrogen supplementation by adding various nitrogen sources at the same concentration calculated based on their nitrogen equivalence (1.92 g/L potassium nitrate (KNO_3_), 1.5 g/L ammonium sulfate ((NH_4_)_2_SO_4_), or 0.67 g/L of urea) into the AP medium; 4) varying the incubation temperature at 15, 20, 25, and 30 °C; 5) varying the initial pH (4.5, 5.5, 6.5, 7.5, or 8.5) of the AP medium; and 6) varying the incubation time at 1, 3, 5, and 7 days. The condition that gave the highest astaxanthin yield was selected for the following experiments.

### Analytical procedures

#### Quantitative analysis of astaxanthin production

Astaxanthin content was quantitatively analyzed using previously described methods [25] with slight modifications. In brief, 50 mg of lyophilized cells were suspended in 5 mL of DMSO (Sigma-Aldrich, USA), ultrasonicated at 37 kHz, 50 °C for 30 min (Elmasonic, E60H model, Germany), and centrifuged at 12.300 × g, for 5 min. The extraction process was repeated until the cell pellet became colorless. The obtained supernatant was analyzed for astaxanthin concentration using an Agilent Cary 60 UV-Vis spectrophotometer at 530 nm against a pure DMSO blank [26]. A standard curve of absorbance versus astaxanthin concentrations was created by dissolving the following concentrations in DMSO: 0, 0.25, 0.5, 1, 2, 4, 6, and 8 μg/ml. The concentration of astaxanthin was calculated using the equation of the astaxanthin standard calibration curve. The results were explained as the mean of three measurements taken in triplicate.

#### Statistical analysis

The obtained results were analyzed using One-way ANOVA, and a multiple comparison test (Duncan’s test) was performed using SPSS Statistic 22.0 Software. A P-value less than 0.05 (*p* < 0.05) was considered statistically significant. Supporting information on statistical analysis is shown in the S1 and S2 Files.

### Genomic DNA sequencing, genome assembly and annotation, and gene analysis

#### Genomic DNA sequencing

The genomic DNA was extracted using the methods previously described [27]. The quality of the DNA was determined using agarose gel electrophoresis and quantified using a NanoDrop One spectrophotometer (Thermo Fisher Scientific, United States). The whole genome was sequenced pair end PE150 on the IlluminaHiseq Xten/Novaseq/MGI2000 platforms at Vishuo Biomedical Pte. Ltd. (Beijing, China).

#### Genome assembly and annotation

The obtained single-end reads were trimmed to remove adapters and low-quality bases using fastp (v0.23.0), and then the optimized data were assembled into contigs using Velvet *de novo* assembler version 1.2.10 [28]. Subsequently, SSPACE (version 3.0) was used to assemble the contigs into scaffolds. Gaps were filled by GapFiller versions 1–10. Gene prediction was performed using Augustus version 3.3 [29]. The program Barrnap (v0.9) and tRNAscan-SE (v1.3.1) were used for determining ribosomal RNA (rRNA) and transfer RNA (tRNAs) in the assembly genome, respectively. The other Non-coding RNAs (ncRNA) were predicted using the mapping Rfam method (v12.2). The coding genes were annotated with National Center for Biotechnology Information (NCBI) nr database by BLAST. Then, gene function prediction was performed by KEGG (Kyoto Encyclopedia of Genes and Genomes) [30] and KofamKOALA tools [31] using the default parameters and all hits. The circular genome mapping was created using Proksee server. The Venn diagram was constructed using the OrthoVenn.

## Results

### Isolation and identification of astaxanthin-producing yeasts

During the study of yeast diversity in flowers, two pigment yeast isolates, TL35-5 and PL61-2, were isolated from marigold flowers (*Tagetes erecta*) and white desert rose flowers (*Plumeria obtusa*) samples collected from Lampang province, Thailand, respectively. In the preliminary screening phase, isolates TL35-5 and PL61-2 displayed a positive spot on the thin-layer chromatography (TLC) silica gel G plate, resembling the astaxanthin standard (with an Rf value of 0.28). High-performance liquid chromatography (HPLC) and liquid chromatography-mass spectrometry (LC-MS) analyses were conducted to confirm this, following previously described methods [16]. The HPLC chromatogram verified the peaks corresponding to the extracted astaxanthin from TL35-5 and PL 61–2, which were identified with a retention time of 2.063. Furthermore, LC-MS analysis employing MS/MS detection in negative scan mode revealed that the astaxanthin extracted from TL35-5 and PL61-2 contained product ions resembling those of the astaxanthin standard.

Additionally, quantitative analysis showed significant intracellular astaxanthin accumulation in both isolates. TL35-5 cultivated in YM broth medium at 25°C for 72 hours had an astaxanthin content and yield of 0.28 ± 0.16 mg/g dry cell weight (DCW) and 2.34 ± 0.0015 mg/L, respectively. Under the same conditions, isolate PL61-2 exhibited an astaxanthin content and yield of 0.058 ± 0.11 mg/g DCW and 0.43 ± 0.0009 mg/L, respectively.

The TL35-5 and PL61-2 isolates were identified as molecular operational taxonomic units (MOTUs) through 26S rRNA (LSU D1/D2 domain) gene sequencing and further assigned to species by comparing them to entries in the NCBI GenBank database using the BLASTn program. The strains TL35-5 and PL61-2 showed 100% identity and were identified as *Rhodotorula paludigena* and *R*. *sampaioana*, respectively. The accession numbers of the LSU D1/D2 domain sequences for strains TL35-5 and PL61-2 submitted to the NCBI/GenBank database were OP547860 and OP547865, respectively.

**Fig 1:**
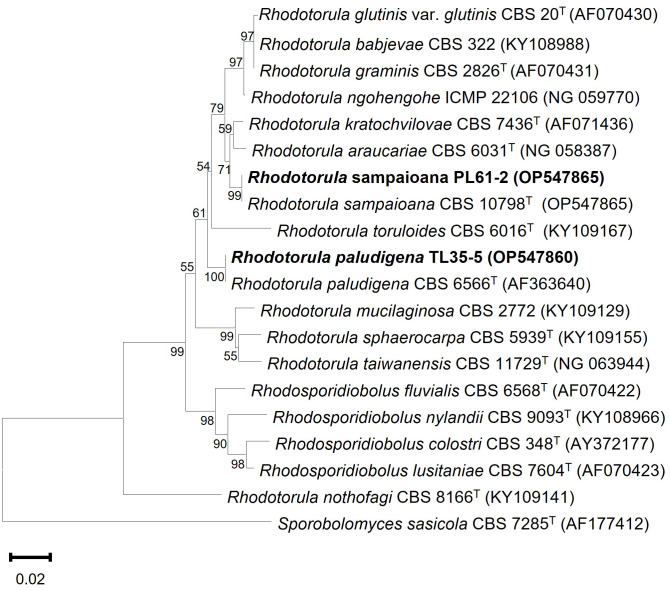
Phylogenetic analysis. Phylogenetic tree of the LSU D1/D2 sequences showing the placement of *R*. *paludigena* TL35-5, *R*. *sampaioana* PL61-2 and the related taxa. *Sporobolomyces sasicola* was used as the outgroup species. Bootstrap values equal to and more than 50%, determined from 1000 replicates are shown next to branches GenBank accession numbers are given in parentheses. Bar 0.20 substitutions per nucleotide position.

The morphological and physiological characteristics of each strain were examined (S1 Fig). TL35-5 cells were ovoid to ellipsoidal in shape, measuring 1.80–4.02 μm in length and 2.90–7.21 μm in width, with predominantly polar budding. The colony cultures appeared raised, round, shiny, butyrous, smooth, and had an entire margin. The streak culture exhibited a red-orange color. The cells of PL61-2 were predominantly ovoid, with a size of 2.68–4.22 μm in length and 3.47–5.45 μm in width. They occurred singly, with polar budding, while the colonies displayed a raised, round morphology with a light peach color, shiny and smooth surface, and entire margin. Neither pseudohyphae nor true mycelium were observed in any of the examined strains. The carbon assimilation characteristics of TL35-5 and PL61-2, compared with their closest related species, are summarized and presented in S1 Table. Phylogenetic analysis using the neighbor-joining (NJ) method with Kimura two-parameter distances based on the D1/D2 domain sequence confirmed the clustering of TL35-5 with *R*. *paludigena* (100% bootstrap values for branch placements), while PL61-2 was clustered with *R*. *sampaioana* (99% bootstrap values for branch positions) (Fig 1).

### Optimization of astaxanthin production of *R*. *paludigena* TL35-5 and *R*. *sampaioana* PL61-2

#### Effects of different carbon sources

**Fig 2:**
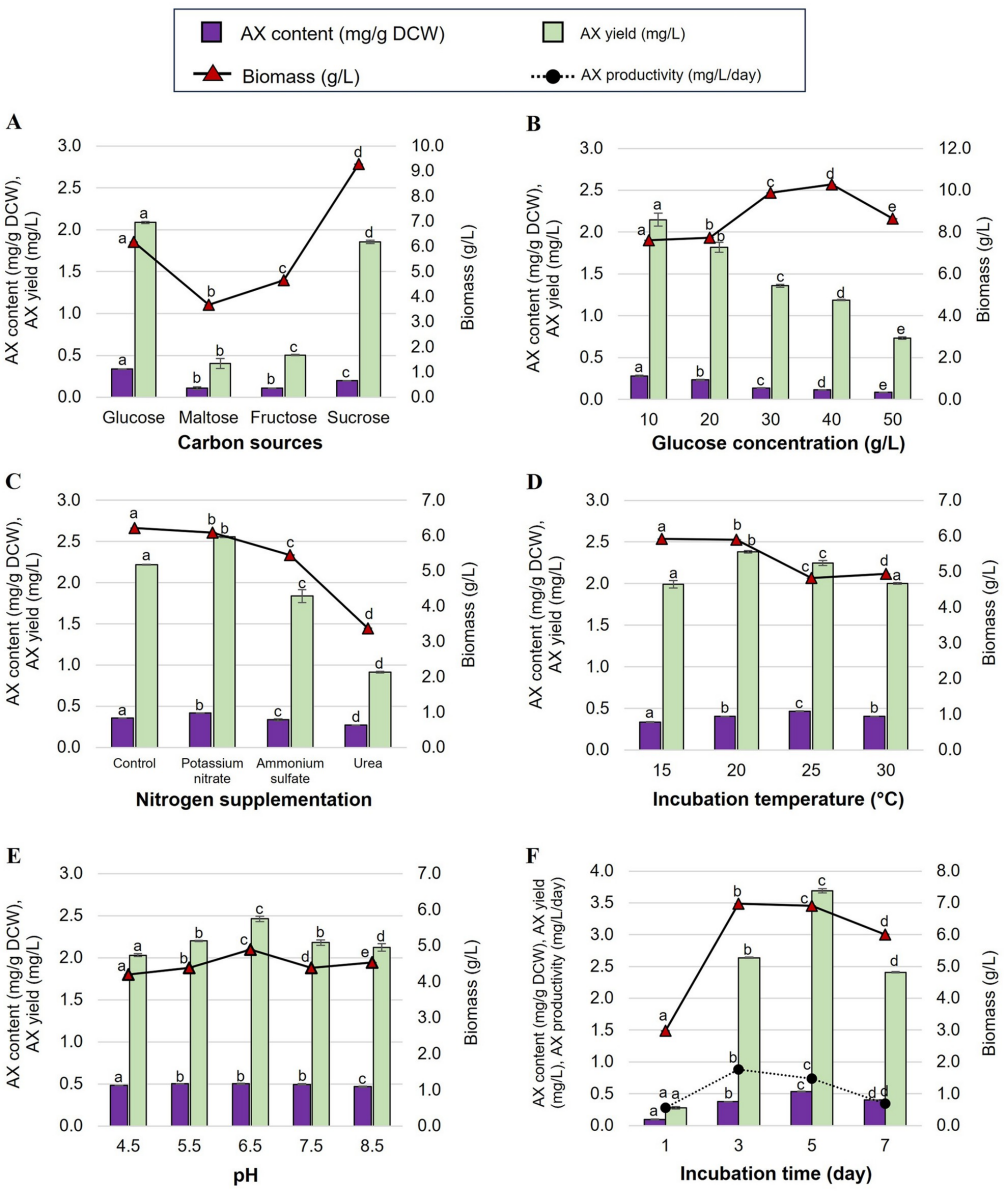
Optimization of astaxanthin production of *R*. *paludigena* TL35-5. Effect of carbon source (A), glucose concentration (B), nitrogen supplementation (C), incubation temperature (D), pH (E), and incubation time (F) on astaxanthin (AX) content, AX yield, biomass, and AX production of *R*. *paludigena* TL35-5. The data are displayed as the mean ± SD, and are derived from triplicate experiments. According to Duncan’s test (*p* < 0.05), the levels that are not linked by the same letter are statistically different.

**Fig 3:**
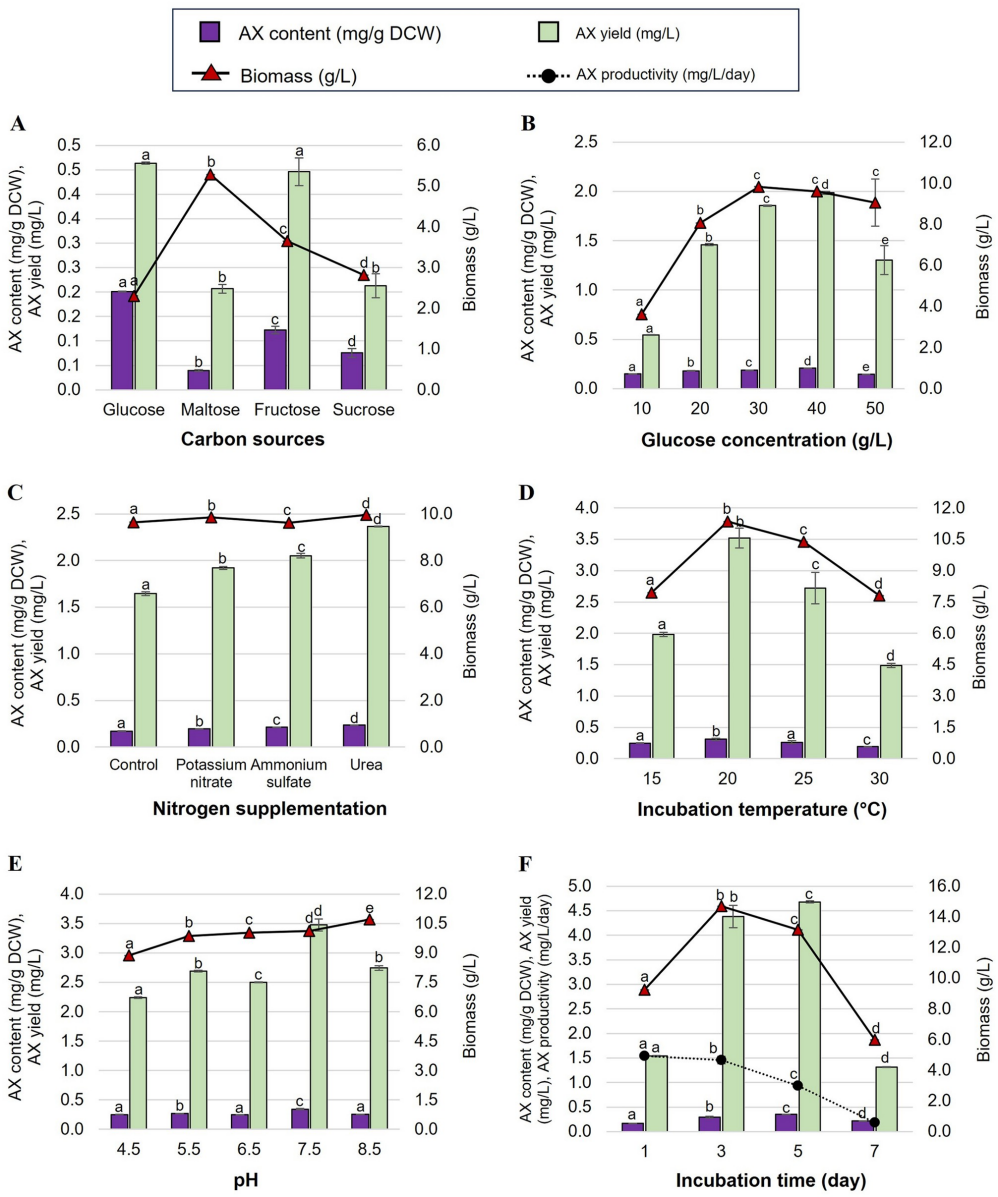
Optimization of astaxanthin production of *R*. *sampaioana* PL61-2. Effect of carbon source (A), glucose concentration (B), nitrogen supplementation (C), incubation temperature (D), pH (E), and incubation time (F) on astaxanthin (AX) content, astaxanthin yield, biomass, and astaxanthin production of *R*. *sampaioana* PL61-2. The data are displayed as the mean ± SD, and are derived from triplicate experiments. According to Duncan’s test (*p* > 0.05), the levels that are not linked by the same letter are statistically different.

The production of astaxanthin in AP medium with the same concentration (10 g/L) of four different carbon sources, namely glucose, maltose, fructose, or sucrose, was examined. The highest astaxanthin content and yield for TL35-5 were 0.338 ± 0.002 mg/g DCW and 2.087 ± 0.011 mg/L, respectively, which were obtained when glucose (10 g/L) was used as the sole carbon source (Fig 2A). Similarly, as shown in Fig 3A, the maximum astaxanthin content and yield for PL61-2 were 0.201 ± 0.001 mg/g DCW and 0.464 ± 0.002 mg/L, respectively, when the yeast was cultured in AP medium supplemented with 40 g/L glucose. Additionally, TL35-5 exhibited the highest biomass (9.281 ± 0.001 g/L) when cultivated in AP medium with sucrose, while PL61-2 achieved the highest biomass (5.283 ± 0.003 g/L) with maltose. However, the focus was primarily on product yield. Therefore, glucose was selected as the optimal carbon source for further optimization of both strains.

The glucose concentration in the AP medium was varied at 10, 20, 30, 40, and 50 g/L to investigate its effect on astaxanthin production. For TL35-5, the optimum concentration for maximum astaxanthin production was found to be 10 g/L of glucose (astaxanthin content of 0.282 ± 0.010 mg/g DCW and astaxanthin yield of 2.149 ± 0.079 mg/L) (Fig 2B). Interestingly, the astaxanthin content and yield of PL61-2 increased and reached their maximum (0.207 ± 0.002 mg/g DCW and 1.987 ± 0.016 mg/L, respectively) when the glucose concentration in the AP medium was increased to 40 g/L (Fig 3B). Therefore, the AP medium containing 40 g/L of glucose was used for further study on astaxanthin production in PL61-2.

#### Effect of nitrogen sources

Nitrogen was identified as one of the most critical factors influencing microbial growth and product production. An examination of the effects of three different nitrogen supplements, namely potassium nitrate, ammonium sulfate, and urea, was conducted. It was observed that the addition of 1.92 g/L of potassium nitrate (KNO_3_) to the AP medium containing 10 g/L glucose significantly increased the highest astaxanthin content and yield of TL35-5 to 0.420 ± 0.001 mg/g DCW and 2.558 ± 0.009 mg/L, respectively, compared to the control AP medium without any nitrogen supplementation (Fig 2C). Additionally, the highest astaxanthin content (0.237 ± 0.0004 mg/g DCW), astaxanthin yield (2.36 ± 0.005 mg/L), and biomass (9.9965 ± 0.008 g/L) of PL61-2 were obtained when cultivated in AP medium supplemented with 0.67 g/L urea (Fig 3C).

#### Effect of temperature

The influence of incubation temperature (15, 20, 25, or 30 °C) on astaxanthin production in TL35-5 and PL61-2 was investigated using their optimized AP medium from the previous study. It was observed that TL35-5 and PL61-2 exhibited the highest astaxanthin yield (2.381 ± 0.015 and 3.521 ± 0.158 mg/L, respectively) when cultured at 20 °C. Therefore, this temperature was selected as the optimal condition for astaxanthin production in both TL35-5 and PL61-2 (Figs 2D and 3D).

#### Effect of pH

The impact of initial pH on astaxanthin production was investigated in the optimized AP medium. The pH was varied to 4.5, 5.5, 6.5, 7.5, or 8.5, and the cultures were incubated at 20 °C and 200 rpm for 7 days. TL35-5 showed the highest astaxanthin yield (2.461 ± 0.033 mg/L) at pH 6.5 (Fig 2E). On the other hand, PL61-2 exhibited maximum astaxanthin production (astaxanthin content of 0.344 ± 0.010 mg/g DCW, astaxanthin yield of 3.477 ± 0.104 mg/L) at pH 7.5 (Fig 3E). These pH values were determined to be the optimal conditions for astaxanthin production in TL35-5 and PL61-2, respectively.

#### Effect of incubation time

The effect of incubation times ranging from 1 to 7 days on astaxanthin accumulation in TL35-5 and PL61-2 was investigated. The TL35-5 had the highest astaxanthin content (0.534 ± 0.004 mg/g DCW) and astaxanthin yield (3.689 ± 0.031 mg/L) when cultivated in optimum AP medium for 5 days (Fig 2F). The PL61-2 also gave the highest content and yield of astaxanthin (0.355 ± 0.001 g/g DCW and 4.680 ± 0.019 mg/L, respectively) on day 5 (Fig 3F). However, the highest astaxanthin productivity of TL35-5 (0.738 ± 0.03 mg/L/day) and PL61-2 (1.461 ± 0.076 mg/L/day) was obtained on day 3. Therefore, the optimal incubation time for astaxanthin production of TL35-5 and PL61-2 was 5 days.

### Genome sequencing, functional annotation, and comparative analysis of protein families

**Fig 4:**
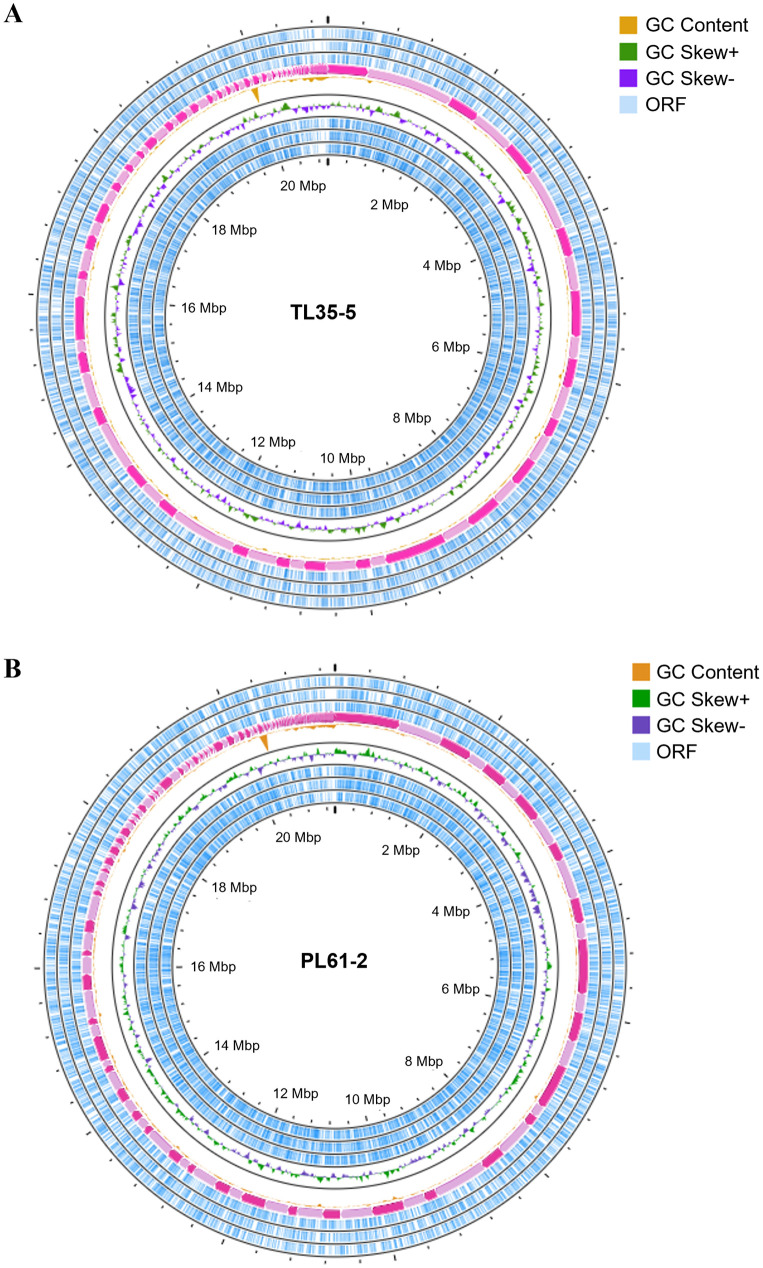
Circular genome maps. *R*. *paludigena* TL35-5 (A) and *R*. *sampaioana* PL61-2 (B). The information is indicated as follows: GC content (orange), GC skew (+) (green), GC skew (−) (purple), and open reading frames (ORFs) (blue).

The genome data of two newly isolated astaxanthin-producing yeasts, *R*. *paludigena* TL35-5 *and R*. *sampaioana* PL61-2, are presented in Table 1. The circular genomic maps of TL35-5 and PL61-2 are shown in Fig 4. TL35-5 has a genome size of 20,982,417 bp with a GC content of 64.20%. It consists of 188 scaffolds with an N50 value of 399,886 bp and an L50 value of 18 bp. The longest and shortest scaffolds are 1,173,495 bp and 329 bp, respectively. TL35-5 genome contains a total of 162 non-coding RNAs, including 9 rRNA, 139 tRNA, and 14 other ncRNA. In strain PL61-2, the estimated genome size is 21,374,169 bp distributed across 291 scaffolds. The assembled genome has a GC content of 64.88%, an N50 length of 339,562 bp, and an L50 length of 23 bp. The longest scaffold in PL61-2 is 892,677 bp, while the shortest scaffold is 360 bp. PL61-2 genome contains 6 rRNA, 171 tRNA, and 10 other ncRNA. The genome sequences of TL35-5 and PL61-2 have been deposited in the NCBI/GenBank database under the BioProject of PRJNA916389 and PRJNA916391, BioSample of SAMN32424969 and SAMN32425014, and GenBank accession numbers JAQRHP000000000 and JAPZQE000000000, respectively.

**Table 1:** General features of *R*. *paludigena* TL35-5 and *R*. *sampaioana* PL61-2 genomes.

Feature	Strains	
	*R*. *paludigena* TL35-5	*R*. *sampaioana* PL61-2
Assembly length (bp)	20,982,417	21,374,169
GC content (%)	64.20	64.88
N50 (bp)	399,886	339,562
L50 (bp)	18	23
Max length (bp)	1,173,495	892,677
Min length (bp)	329	360
Number of contigs	243	440
Number of scaffolds	188	291
Predicted protein-coding gene	6,789	6,802
*RNA*		
Total ncRNA	162	187
rRNA	9	6
tRNA	139	171
Other ncRNA	14	10

Protein-coding genes were also predicted and annotated in this study. Out of the 6,789 predicted genes in the TL35-5 genome, 6,316 genes (93.03%) were annotated using the NCBI Nr databases based on sequence homology. Additionally, 2,367 genes (34.87%) were annotated with the KEGG database. In PL61-2 genome, 6,803 genes were predicted, of which 6,218 genes (91.40%) and 2,319 genes (34.09%) were annotated with their homologs in the Nr database and KEGG metabolic pathway, respectively. The annotated genes of TL35-5 and PL61-2 genomes mapped to the KEGG metabolism pathway are shown in S2 Fig. The top three KEGG pathways for annotated genes in TL35-5 and PL61-2 genomes are "Genetic information processing," "Cellular processes," and "Carbohydrate metabolism."

**Fig 5:**
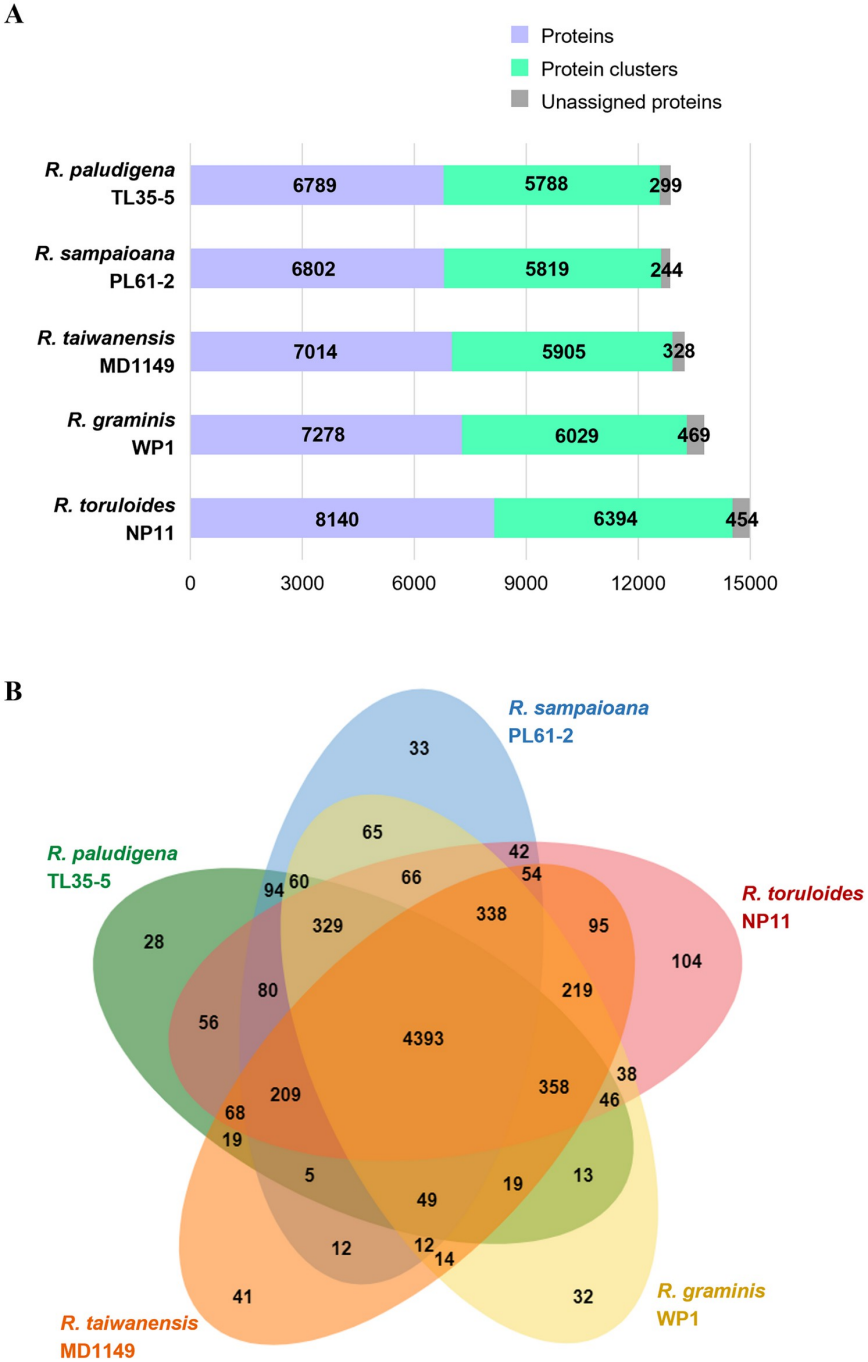
Comparative genomic analysis. Whole-genome comparison between *Rhodotorula paludigena* TL35-5, *R*. *sampaioana* PL61-2, *R*. *toruloides* NP11, *R*. *graminis* WP1, and *R*. *taiwanensis* MD1149. The number of proteins, protein families, and unassigned proteins of each *Rhodotorula* species (A). Venn diagram representation of shared or unique protein clusters (B).

Furthermore, a comparative genomic analysis was performed between TL35-5, PL61-2, and three other well-known *Rhodotorula* species: *R*. *toluroides* NP11, *R*. *graminis* WP1, and *R*. *taiwanensis* MD1149, to identify species-specific protein families. As shown in Fig 5A, 6,394, 6,029, 5,905, 5,819, and 5,788 protein families were identified for NP11, WP1, MD1149, PL61-2, and TL35-5, respectively. Among them, 28, 33, 104, 32, and 41 protein families were found to be species-specific for PL35-5, PL61-2, NP11, WP1, and MD1149, respectively (Fig 5B).

### Identification of a gene involved in the astaxanthin biosynthesis

Due to the potential of *R*. *paludigena* TL35-5 and *R*. *sampaioana* PL61-2 as astaxanthin-producing yeasts, we conducted an analysis of genes involved in astaxanthin biosynthesis in their genomes. This study aimed to identify all the probable essential genes required for astaxanthin production in TL35-5 and PL61-2, which are listed in Table 2. Interestingly, we discovered the presence of the astaxanthin synthetase gene (*CrtS*) and the cytochrome P450 reductase (*CrtR*) gene in the genomes of TL35-5 and PL61-2. These genes are known to be key and conserved in *X*. *dendrorhous* and are involved in astaxanthin synthesis.

**Table 2:** Putative gene coding clusters for the biosynthesis of terpenoid backbone and astaxanthin in *R*. *paludigena* TL35-5 and *R*. *sampaioana* PL61-2 genomes.

Pathways	TL35-5 Scaffold ID (gene ID)	PL61-2 Scaffold ID (gene ID)	Most similar known gene	KEGG orthologs (KO) number, EC number
Terpenoid backbone biosynthesis	19 (g3176)	9 (g1233)	*ACAT* (Acetyl-CoA acetyltransferase)	K00626, EC:2.3.1.9
	13 (g2244)	101 (g6434)	*HMGCS* (Hydroxymethylglutaryl-CoA synthase)	K01641, EC:2.3.3.10
	96 (g6732)	12 (g1591)	*HMGCR* (Hydroxymethylglutaryl-CoA reductase)	K00021, EC:1.1.1.34
	19 (g3064)	50 (g5011)	*MVD* (Mevalonate kinase)	K00869, EC:2.7.1.36
	19 (g2943)	9 (g1202)	*PMK* (Phosphomevalonate kinase)	K00938, EC:2.7.4.2
	73 (g6580)	49 (g4891)	*DPMDC* (Diphosphomevalonate decarboxylase)	K01597, EC:4.1.1.33
	19 (g3118)	9 (g1180)	*IDI* (Isopentenyl-diphosphate delta isomerase)	K01823, EC:5.3.3.2
	2 (g424)	48 (g4874)	*GGDPS* (Geranylgeranyl diphosphate synthase)	K00804, EC:2.5.1.1 2.5.1.10 2.5.1.29
	10 (g1845)	22 (g2914)	*FDPS* (Farnesyl diphosphate synthase)	K00787, EC:2.5.1.1 2.5.1.10
	9 (g1700)	17 (g2273)	*CrtE* (Geranylgeranyl pyrophosphate synthase)	K05355, EC:2.5.1.82 2.5.1.83
Astaxanthin biosynthesis	5 (g1023)	15 (g2088)	*CrtYB* (Phytoene synthase/ lycopene β-cyclase)	K17841, EC:2.5.1.32 5.5.1.19
	5 (g1025)	15 (g2086)	*CrtI* (Phytoene desaturase)	K15745, EC:1.3.99.30
	26 (g3929)	47 (g4812)	*CrtS* (Astaxanthin synthetase, β-carotene 4-ketolase/3-hydroxylase)	K23037, EC:1.14.99.63 1.14.15.24 1.14.99-
	33 (g4648)	2 (g477)	*CrtR* (Cytochrome P450)	K14338, EC:1.14.14.1 1.6.2.4

**Fig 6:**
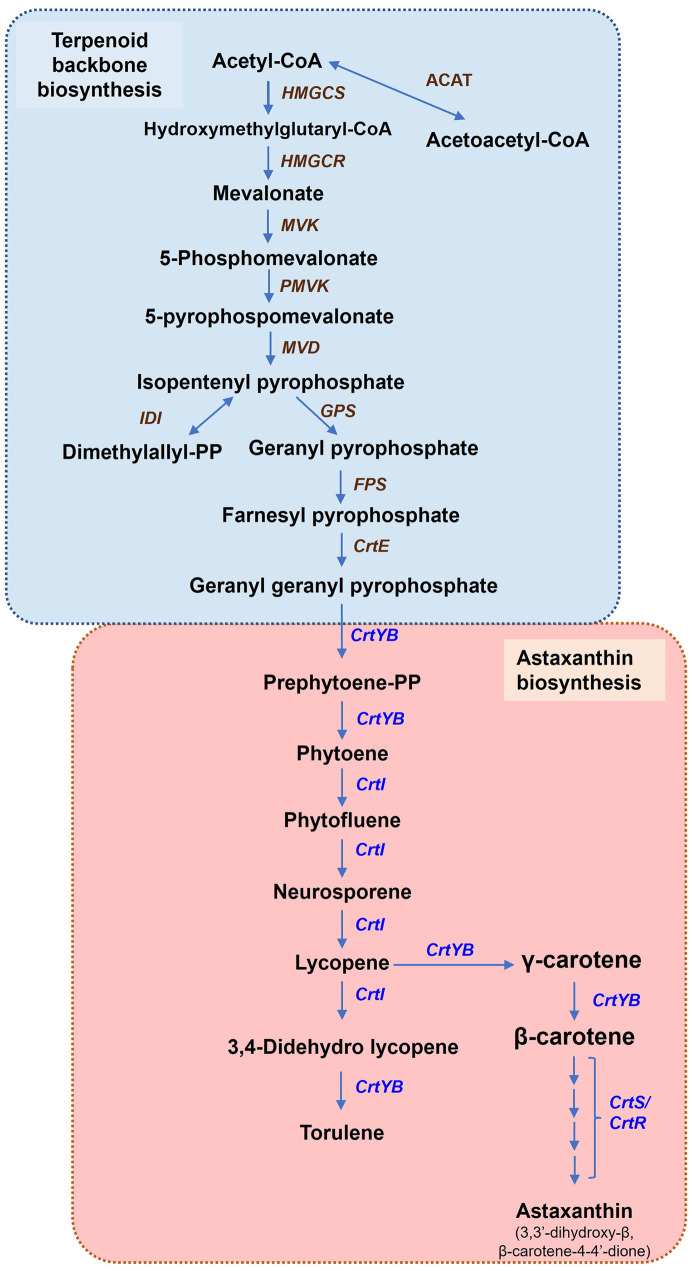
Schematic of the probable astaxanthin biosynthetic pathways in *R*. *paludigena* TL35-5 and *R*. *sampaioana* PL61-2. Metabolites are shown in bold, while the genes encoding enzymes responsible for relative bioconversions are indicated in italics at corresponding arrows.

Based on the gene data obtained, we propose a possible bioconversion process of TL35-5 and PL61-2, starting from the geranylgeranyl pyrophosphate obtained from terpenoid backbone synthesis, leading to the production of astaxanthin (as illustrated in Fig 6). Similar to other carotenoid biosynthesis yeasts, the biosynthesis mechanism begins with the synthesis of the precursor, geranylgeranyl pyrophosphate (*GGPP*), through the mevalonate pathway of terpenoid backbone synthesis. Subsequently, two putative key enzymes, phytoene desaturase (*CrtI*) (EC:1.3.99.30) and the bifunctional phytoene synthase/lycopene cyclase (*CrtYB*) (EC:2.5.1.32 5.5.1.19), play active roles in carotenoid biosynthesis, resulting in the formation of β-carotene and torulene. Finally, the synthesis of astaxanthin takes place with the involvement of the key enzyme astaxanthin synthetase and its helper enzyme, cytochrome P450 reductase (encoded by the *CrtS* and *CrtR* genes, respectively). These enzymes facilitate the formation of astaxanthin and other xanthophyll derivatives.

## Discussion

Several *Rhodotorula* species, pigmented basidiomycetous yeasts, have been identified as biotechnologically significant carotenoid producers. The carotenoid profile of most yeast species belonging to the genus *Rhodotorula* primarily consists of β-carotene, γ-carotene, torulene, and torularhodin. Interestingly, some *Rhodotorula* species, such as *Rhodotorula toruloides* VN1 [15, 32], and *Rhodotorula* sp. CP72-2 [16], have been reported as natural astaxanthin producers, similar to the red yeast *Xanthophyllomyces dendrorhous*.

In this study, two pigmented yeasts belonging to the Basidiomycota phylum and the genus *Rhodotorula* were examined: *Rhodotorula paludigena* strain TL35-5 and *Rhodotorula sampaioana* strain PL61-2. TL35-5 was isolated from Marigold flowers (*Tagetes erecta*), while PL61-2 was isolated from white desert rose flowers (*Plumeria obtusa*). *R*. *paludigena* has previously been identified as a marine yeast frequently isolated from various marine samples, including deep-sea sediment [33], mangrove soil, and mangrove water [19, 34]. However, *R*. *paludigena* is not limited to marine habitats as it has also been found in other biological samples such as plant nectar [35], and castor beans [36]. *R*. *sampaioana* was originally isolated from two different habitats: subsurface waters of Lake Negra in Argentina and the gut of a xylophagous termite in India [37]. Surprisingly, our preliminary study revealed that both TL35-5 and PL61-2 have the ability to produce astaxanthin, which is a rare and unique characteristic among *Rhodotorula* yeasts. TL35-5 exhibited an astaxanthin content and yield of 0.28 ± 0.16 mg/g dry cell weight (DCW) and 2.34 ± 0.0015 mg/L, respectively. Meanwhile, PL61-2 produced astaxanthin with a content and yield of 0.058 ± 0.11 mg/g DCW and 0.43 ± 0.0009 mg/L, respectively. Although *R*. *paludigena* has been acknowledged for its production of carotenoids, single-cell oil, and biosurfactants [36, 38, 39], research on its capacity to synthesize astaxanthin remains limited [40]. On the other hand, the biotechnological potential of *R*. *sampaioana* has not been reported, making this the first study on astaxanthin production from *R*. *sampaioana* specie.

Astaxanthin production, which is a secondary metabolite produced by pigmented yeast, is affected by various cultivation factors such as media composition, environmental conditions, and cultivation parameters [5]. Nutrient availability and composition in the cultivation medium play a crucial role in astaxanthin biosynthesis. Carbon and nitrogen sources, minerals, vitamins, and other micronutrients must be carefully balanced to support yeast growth and stimulate astaxanthin production. Furthermore, temperature and pH conditions, light intensity and wavelength, and agitation and aeration levels in the fermentation vessel profoundly influence astaxanthin production. To maximize astaxanthin production in *R*. *paludigena* TL35-5 and *R*. *sampaioana* PL61-2, we optimized the medium composition and environmental factors using the one-factor-at-a-time method. Among the tested carbon sources, glucose was found to be the optimal carbon source for astaxanthin production in both TL35-5 and PL61-2. Their astaxanthin content and yield reached their maximum when cultured in AP medium with glucose (10 g/L) as the sole carbon source. This finding confirms that glucose is the best carbon source for astaxanthin production. Although the highest biomass for TL35-5 and PL61-2 was achieved when sucrose and maltose were used as carbon sources, respectively, it is important to note that astaxanthin production, being a secondary metabolite, does not necessarily require optimal cell growth conditions. Previous studies have shown that astaxanthin biosynthesis depends on various environmental stressors that are not conducive to optimal cell growth [41]. In contrast, Guo et al. [42] reported that the maximum cell biomass and astaxanthin yield (7.6 g/L and 3.22 mg/L, respectively) of *Phaffia rhodozyma* CGMCC As 2.1557 were both observed in a sucrose medium.

In addition, the concentration of glucose has also been identified as an important factor in astaxanthin production. The optimal glucose concentration in AP medium for TL35-5 astaxanthin production was found to be 10 g/L. Under these conditions, the content and yield of astaxanthin reached their maximum values at 2.282 ± 0.010 mg/g DCW and 2.149 ± 0.079 mg/L, respectively. However, when the glucose concentration was increased to 50 g/L, astaxanthin production decreased while cell biomass increased. This observation suggests that the fermentation process, occurring under aerated conditions with high glucose concentrations, may be suppressed due to the Crabtree effect. The Crabtree effect can inhibit aerobic metabolism in yeast, including catabolism and respiration [43, 44], resulting in some yeast strains fermenting glucose into ethanol under aerobic conditions [45] or producing more biomass. Additionally, the higher osmotic pressure associated with increased glucose concentration may also contribute to these effects. For PL61-2, increasing the glucose concentration from 10 to 40 g/L in the AP medium led to a 3.65-fold increase in astaxanthin yield and a 2.65-fold increase in biomass. Tran et al. [15] reported that the optimal glucose concentration for astaxanthin production in *R*. *toruloides* is 83.74 g/L.

Among the three nitrogen sources evaluated, it was found that AP medium supplemented with 1.92 g/L potassium nitrate, an inorganic nitrogen source, was the most favorable for TL35-5 astaxanthin production. Interestingly, our findings revealed that under nitrogen-excess conditions, TL35-5 cell proliferation decreased while astaxanthin production increased. On the other hand, the addition of 0.67 g/L urea, an organic nitrogen source, was identified as the optimal nitrogen supplementation for astaxanthin production in PL61-2. It has been reported that nitrogen limitation can effectively induce astaxanthin production in the alga *H*. *pluvialis* [46]. Some studies have also highlighted low-cost nitrogen sources like corn steep liquor as alternatives for astaxanthin production in *P*. *rhodozyma* D3 [47]. Additionally, yeast extract, which is an organic nitrogen source, has been found to be the optimal nitrogen source for astaxanthin production in *P*. *rhodozyma* CGMCC As 2.1557 [42]. For *P*. *rhodozyme* 7B12, the optimal nitrogen sources for astaxanthin production were determined to be 0.28 g/L (NH_4_)_2_SO_4_, 0.49 g/L KNO_3_, and 1.19 g/L beef extract, resulting in the highest astaxanthin yield of 7.71 mg/L under these optimal conditions [48].

Temperature is a crucial factor that influences all living organisms and plays a role in regulating growth and metabolite biosynthesis. Changes in temperature can significantly impact enzymatic activities, thereby affecting cell growth and astaxanthin production [41]. However, the effect of temperature on carotenoid production varies depending on the strain [49, 50]. Hayman et al. [51] reported that temperature influences the regulation of two key enzymes involved in carotenoid biosynthesis, β-carotene synthase and torulene synthase, in *R*. *glutinis*. However, there is limited research on the effect of temperature on astaxanthin production. In our study, we found that the optimal temperature for astaxanthin production in *R*. *paludigena* TL35-5 and *R*. *sampaioana* PL61-2 was 20 °C. This finding aligns with Polulyakh et al. [52], who reported that the optimal temperature for astaxanthin biosynthesis in *P*. *rhodozyma* is also 20 °C. Under these conditions, *P*. *rhodozyma* produced a higher amount of astaxanthin, accounting for up to 85% of total carotenoids. Johnson and Lewis [53] similarly reported that the temperature range of 20–22 °C was optimal for *X*. *dendrorhous* astaxanthin production. Additionally, the other *Rhodotorula* species showed the highest astaxanthin synthesis at 25 °C [16, 40].

The initial pH value is a crucial environmental factor that significantly affects the final yield of astaxanthin production. Similarly, the production of astaxanthin in *R*. *paludigena* TL35-5 and *R*. *sampaioana* PL61-2 is influenced by the pH of the medium. The highest astaxanthin content and yield for TL35-5 and PL61-2 were achieved when cultured in the optimal AP medium with initial pH values of 6.5 and 7.5, respectively. According with Phuengjayaem et al. [40], the optimal pH condition for astaxanthin production of *R*. *paludigena* SP9-15 was 7.5. These results suggest that an alkaline environment (pH 6.5–7.5) promotes astaxanthin synthesis in both *Rhodotorula* species. In contrast, *Rhodotorula* sp. CP72-2 showed optimal astaxanthin production in an acidic environment (pH 4.5). These findings indicate that the optimal pH for astaxanthin production in *Rhodotorula* species is strain-dependent, similar to *X*. *dendrorhous* [41]. For *X*. *dendrorhous* UCD 67–210, the optimal pH for growth and astaxanthin production was 4.5 [53]. In the case of *X*. *dendrorhous* J4-3, a mutant strain, the optimal pH for astaxanthin biosynthesis was 5.0, while the optimum pH for growth was 6.0 [54]. According to Ambati et al. [5], optimal temperature and pH ranges for yeast growth and astaxanthin synthesis vary depending on the yeast strain and cultivation conditions but typically fall within specific ranges conducive to both growth and astaxanthin accumulation.

Aside from medium composition and fermentation conditions, the incubation period is another crucial factor that influences pigment synthesis. In various yeast species, carotenoid production typically initiates in the late logarithmic phase and continues into the stationary phase. In this study, the patterns of astaxanthin biosynthesis in TL35-5 and PL61-2 were similar. On the first day of incubation, low levels of astaxanthin were detected. The astaxanthin yields on day 3 increased by 9-fold and 2-fold compared to the first day in TL35-5 and PL61-2, respectively. Subsequently, the astaxanthin yield slightly increased until it reached its peak on day 5 and then gradually decreased. Although the results indicated that the highest astaxanthin yields and biomass for TL35-5 and PL61-2 were obtained on day 5 (120 hours), similar to other previous studies on carotenoid biosynthesis [55, 56], the astaxanthin productivities (mg/L/day) were lower than those obtained on day 3. High astaxanthin productivity is a critical characteristic from a practical production perspective. Therefore, the optimal cultivation period for TL35-5 and PL61-2 was determined to be three days of incubation. This finding reveals that both the cultural medium composition and fermentation conditions affect the growth and astaxanthin production of TL35-5 and PL61-2. However, optimizing all parameters that influence astaxanthin production allows them to reach their maximum production capacity.

In this present study, the maximum astaxanthin yield produced by TL35-5 and PL61-2 under the optimum conditions was 3.689 ± 0.031 and 4.680 ± 0.0.019 mg/L, respectively, which surpasses some previously reported yields. For example, *Spirulina platensis* produced 0.038 mg/L astaxanthin [56], *X*. *dendrorhous* yielded 0.64 mg/L [57], *R*. *toruloides* (wild strain) produced 0.93 mg/L [15], *Rhodotorula* sp. CP72-2 reached 4.13 mg/L [16], and *R*. *paludigena* SP9-15 produced 6.565 ± 0.238 mg/L [40]. Additionally, the mutant strain G17 of *R*. *toruloides* produced 3.02 mg/L astaxanthin when the medium composition was optimized [58]. These findings indicate that the two newly isolated pigmented yeast strains, *R*. *paludigena* TL35-5 and *R*. *sampaioana* PL61-2, have the potential to be natural astaxanthin-producing yeasts.

Here, we present the genome sequences of two newly isolated astaxanthin-producing yeasts, *R*. *paludigena* TL35-5 and *R*. *sampaioana* PL61-2, and analyze the essential genes to provide evidence of their astaxanthin biosynthesis capabilities. The genome size and GC content (%) of TL35-5 (20,982,417 bp and 64.20%) are consistent with *R*. *paludigena* CM33, which had a genome length of 20,657,327 bp and a GC content of 64.3%. Additionally, we successfully assembled the genome of *R*. *sampaioana* PL61-2, which consists of 21,374,169 bp with a GC content of 64.88%. Comparative genomic analysis revealed a close relationship between the genome GC content of PL61-2 (64.88%) and TL35-5 (64.20%), while the predicted number of protein-coding genes in PL61-2 (6,802) is slightly higher than that in TL35-5 (6,789). The number of annotated genes mapped to each KEGG metabolism pathway in TL35-5 and PL61-2 genomes is quite similar. Based on the functional annotation of the genome, we identified several key genes involved in astaxanthin biosynthesis and terpenoid backbone biosynthesis. The following is a summary of the candidate genes: 1) terpenoid backbone biosynthesis, including *ACAT* (Acetyl-CoA acetyltransferase), *HMGCS* (Hydroxymethylglutaryl-CoA synthase), *HMGCR* (Hydroxymethylglutaryl-CoA reductase), *MVD* (Mevalonate kinase), *PMK* (Phosphomevalonate kinase), *DPMDC* (Diphosphomevalonate decarboxylase), *IDI* (Isopentenyl-diphosphate delta isomerase), *GGDPS* (Geranylgeranyl diphosphate synthase), and *FDPS* (Farnesyl diphosphate synthase); 2) Astaxanthin biosynthesis, including *CrtE* (Geranylgeranyl pyrophosphate synthase), *CrtYB* (Phytoene synthase/lycopene β-cyclase), *CrtI* (Phytoene desaturase), *CrtS* (β-carotene 4-ketolase/3-hydroxylase), and *CrtR* (Cytochrome P450 reductase).

Astaxanthin is a metabolite derived from zeaxanthin and canthaxanthin, containing hydroxyl and ketone functional groups. In *X*. *dendrorhous*, astaxanthin is synthesized from β-carotene through the action of the astaxanthin synthetase (*CrtS*) gene, which exhibits both ketolase and hydroxylase activities [4, 59]. Additionally, the cytochrome P450 reductase (*CrtR*) serves as an auxiliary enzyme [60]. Unlike in bacteria and microalgae, the *CrtZ* gene (also known as *CrtR-b* or *Chy*) is responsible for β-carotene hydroxylation, while the *CrtW* gene (also known as *BKT*) is responsible for β-carotenoid ketolation [61]. A recent study identified putative candidate genes associated with astaxanthin biosynthesis, including *CrtE*, *CrtYB*, *CrtI*, *CrtS*, *CrtR*, *CrtW*, *CrtO*, and *CrtZ*, in *Rhodotorula* sp. CP72-2 [16] and *R*. *paludigena* SP9-15 [40].

Our findings suggest that the putative astaxanthin synthesis pathway in *R*. *paludigena* TL35-5 and *R*. *sampaioana* PL61-2 follows a three-step process, similar to *X*. *dendrorhous*, a commercially significant astaxanthin producer [62]. The first step involves the production of isopentenyl pyrophosphate (IPP) from acetyl-CoA in the mevalonate (MVA) pathway, which is part of the terpenoid backbone biosynthesis pathway. IPP is then used for the synthesis of geranylgeranyl pyrophosphate (GGPP). Notably, IPP can be synthesized naturally through either the MVA or the methylerythritol phosphate (MEP) pathways. The MVA pathway is found in fungi, plants (cytosol), and archaea, while the MEP pathway is utilized by bacteria, algae, and higher plants [63]. In the second step, two molecules of geranylgeranyl pyrophosphate are condensed to form phytoene by the enzyme CrtYB, which catalyzes four desaturation steps. In certain fungal species, the *CrtB* and *CrtY* genes are fused, resulting in a bifunctional protein with lycopene cyclase and phytoene synthase activities [64, 65]. Subsequently, the enzymes CrtYB and CrtI cyclize to form β-ionone groups. At this point, the pathway diverges into two branches: the formation of β-carotene and its xanthophyll derivatives, and the synthesis of torulene. The final step involves the production of astaxanthin and other xanthophyll derivatives, such as zeaxanthin, canthaxanthin, and violaxanthin. Astaxanthin synthetase (CrtS) catalyzes the last steps of astaxanthin production from β-carotene, exclusively utilizing the reductase activity of cytochrome P450 reductase (encoded by the *CrtR* gene) as an electron donor [66].

These findings offer valuable resources for advancing the biotechnological applications of *R*. *paludigena* TL35-5 and *R*. *sampaioana* PL61-2. While the genomes of these yeast strains contain the key genes, *CrtS* and *CrtR*, involved in astaxanthin production, limited knowledge exists regarding their functions, as well as the biosynthesis and regulation processes in *Rhodotorula* yeast species. Therefore, conducting *in vivo* functional characterization of these genes remains a crucial prerequisite for future research.

## Supporting information

S1 Fig The vegetative cells morphology under light microscopy (100X magnification) (A, D), colony characteristics on 5% malt extract agar (B, E), and Dalmau slide culture on corn meal agar under light microscopy (100X magnification) (C, F) of *R*. *paludigena* TL35-5 and *R*. *sampaioana* PL61-2, respectively.
(TIF)

S2 Fig The number of annotated genes of (A) TL35-5 and (B) PL61-2 mapped to the KEGG functional category. (TIF)

S1 Table Carbon assimilation characteristics of *R*. *paludigena* TL35-5 and *R*. *sampaioana* PL61-2 compared with their closely related species, *Rhodotorula paludigena* CBS6566^T^ [17] and *Rhodotorula sampaioana* CRUB1124^T^ [37]. (DOCX)

S1 File The supporting results of the statistical analysis on astaxanthin content, astaxanthin yield, and biomass of *R*. *paludigena* TL35-5. (DOCX)

S2 File The supporting results of the statistical analysis on astaxanthin content, astaxanthin yield, and biomass of *R*. *sampaioana* PL61-2. (DOCX)
