# Supplementary figures and images for: Comparative genomic analysis and optimization of astaxanthin production of Rhodotorula paludigena TL35-5 and Rhodotorula sampaioana PL61-2
Source: PLoS One. 2024 Jul 12;19(7):e0304699. doi: 10.1371/journal.pone.0304699 (PMC11244826; doi:10.1371/journal.pone.0304699)

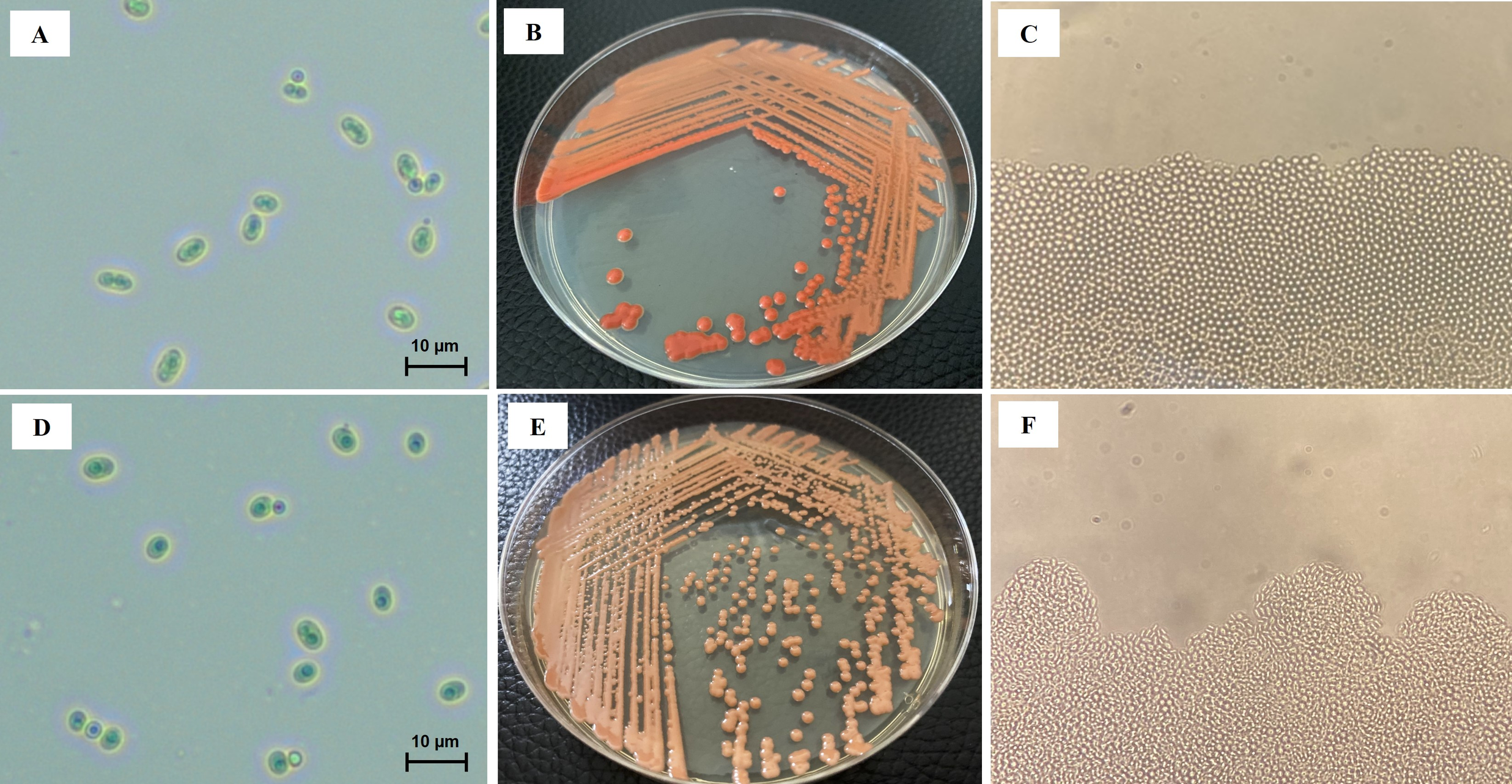

Supplement: S1 Fig — The vegetative cells morphology under light microscopy (100X magnification) (A, D), colony characteristics on 5% malt extract agar (B, E), and Dalmau slide culture on corn meal agar under light microscopy (100X magnification) (C, F) of R. paludigena TL35-5 and R. sampaioana PL61-2, respectively. (TIF) [file pone.0304699.s001.tif]

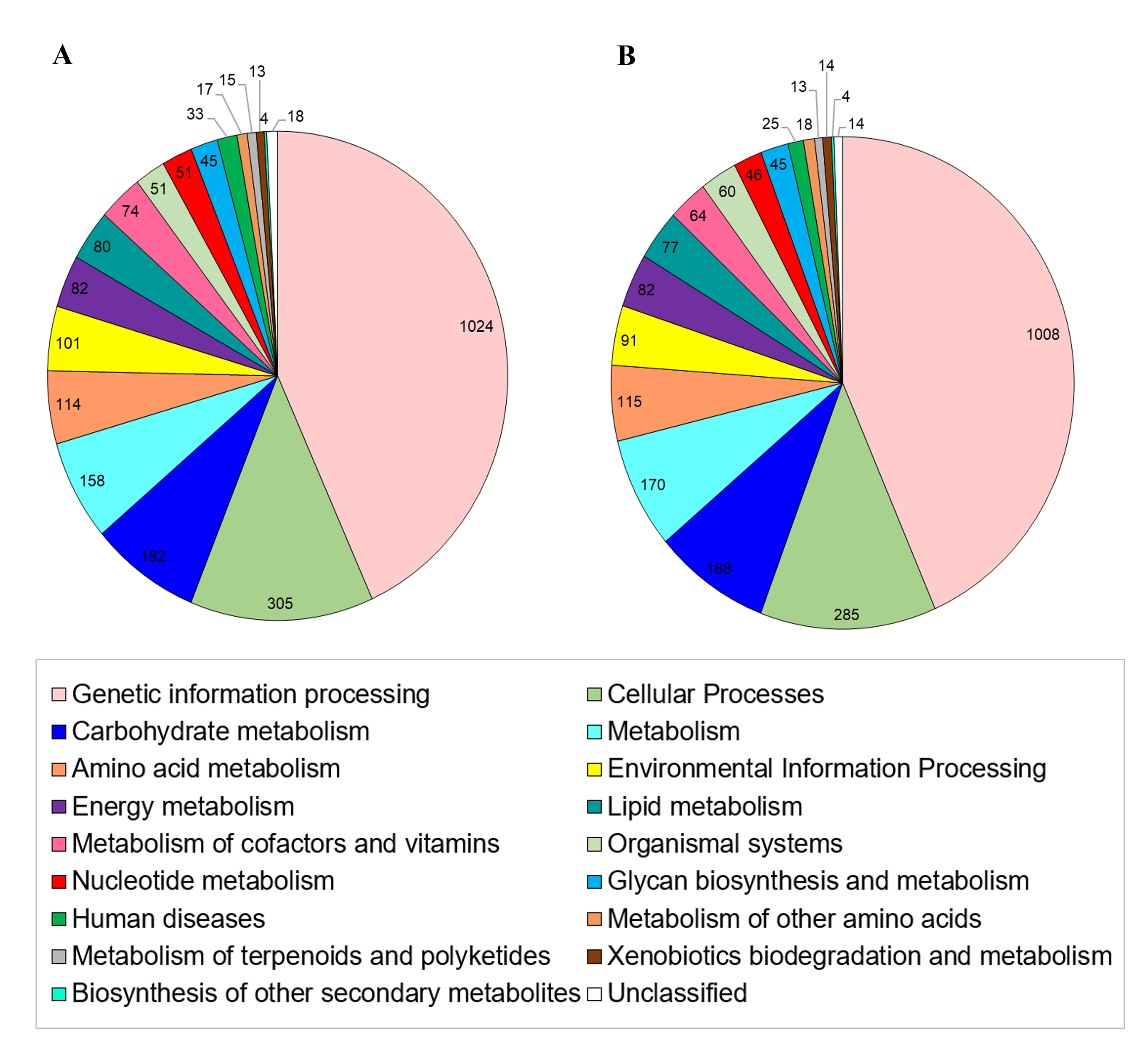

Supplement: S2 Fig — (TIF) [file pone.0304699.s002.tif]
