# Supplementary material for: Comparative genomic analysis and optimization of astaxanthin production of Rhodotorula paludigena TL35-5 and Rhodotorula sampaioana PL61-2
Source: PLoS One. 2024 Jul 12;19(7):e0304699. doi: 10.1371/journal.pone.0304699 (PMC11244826; doi:10.1371/journal.pone.0304699)
Supplement: S1 Table — (DOCX) [file pone.0304699.s003.docx]

| Characteristics | *Rhodotorula paludigena* TL35-5 | *Rhodotorula paludigena* CBS6566^T^ | *Rhodotorula sampaioana*  PL61-2 | *Rhodotorula sampaioana* CRUB1124^T^ |
| --- | --- | --- | --- | --- |
| D-Galactose | + | + | + | + |
| Cycloheximide (actidione) | + | + | - | - |
| D-Saccharose (sucrose) | + | + | + | + |
| N-acetyl-glucosamine | - | ND | - | - |
| Lactic acid | - | - | - | - |
| L-Arabinose | - | +/S | - | - |
| D-Cellobiose | - | + | W | + |
| D-Raffinose | + | + | + | + |
| D-Maltose | + | + | - | - |
| D-Trehalose | + | + | + | + |
| Potassium 2-Ketogluconate | + | ND | + | + |
| Methyl-α-D-Glucopyranoside | - | V | - | ND |
| D-Mannitol | + | + | + | + |
| D-Lactose (bovine origin) | - | - | - | - |
| Inositol | - | - | - | - |
| D-Sorbitol | + | + | + | + |
| D-Xylose | + | + | + | + |
| D-Ribose | + | + | - | - |
| Glycerol | + | + | + | + |
| L-Rhamnose | W | -/W | - | - |
| Palatinose | + | ND | - | ND |
| Erythritol | - | - | - | - |
| D-Melibiose | - | - | - | - |
| Sodium glucuronate | - | ND | - | - |
| D-Melezitose | - | - | - | +/S |
| Potassium gluconate | + | + | + | + |
| Levulinic acid | - | ND | - | ND |
| D-Glucose | + | + | + | + |
| L-Sorbose | - | +/S | - | -/S |
| Glucosamine | - | - | - | - |
| Esculin ferric citrate | + | ND | + | ND |

+, positive; -, negative; ND, not determined; W, weak; V, variable; S, slow
