# Supplementary material for: Comparative genomic analysis and optimization of astaxanthin production of Rhodotorula paludigena TL35-5 and Rhodotorula sampaioana PL61-2
Source: PLoS One. 2024 Jul 12;19(7):e0304699. doi: 10.1371/journal.pone.0304699 (PMC11244826; doi:10.1371/journal.pone.0304699)
Supplement: S1 File — (DOCX) [file pone.0304699.s004.docx]

The effect of different carbon sources on astaxanthin content, astaxanthin yield and biomass of *R. paludigena* TL35-5

**ANOVA**

|  |  | Sum of Squares | df | Mean Square | F | Sig. |
| --- | --- | --- | --- | --- | --- | --- |
| Astaxanthin content | Between Groups | .105 | 3 | .035 | 481.499 | .000 |
|  | Within Groups | .001 | 8 | .000 |  |  |
|  | Total | .106 | 11 |  |  |  |
| Astaxanthin yield | Between Groups | 7.001 | 3 | 2.334 | 2218.442 | .000 |
|  | Within Groups | .008 | 8 | .001 |  |  |
|  | Total | 7.010 | 11 |  |  |  |
| Biomass | Between Groups | 53.881 | 3 | 17.960 | 555475.258 | .000 |
|  | Within Groups | .000 | 8 | .000 |  |  |
|  | Total | 53.881 | 11 |  |  |  |

**Astaxanthin content**

Duncan

| Carbon source | N | Subset for alpha = 0.05 | | |
| --- | --- | --- | --- | --- |
|  |  | 1 | 2 | 3 |
| Fructose | 3 | .10833 |  |  |
| Maltose | 3 | .10933 |  |  |
| Sucrose | 3 |  | .19967 |  |
| Glucose | 3 |  |  | .33767 |
| Sig. |  | .889 | 1.000 | 1.000 |

Means for groups in homogeneous subsets are displayed.

a Uses Harmonic Mean Sample Size = 3.000.

**Astaxanthin yield**

Duncan

| Carbon source | N | Subset for alpha = 0.05 | | | |
| --- | --- | --- | --- | --- | --- |
|  |  | 1 | 2 | 3 | 4 |
| Maltose | 3 | .40267 |  |  |  |
| Fructose | 3 |  | .50400 |  |  |
| Sucrose | 3 |  |  | 1.85333 |  |
| Glucose | 3 |  |  |  | 2.08733 |
| Sig. |  | 1.000 | 1.000 | 1.000 | 1.000 |

Means for groups in homogeneous subsets are displayed.

a Uses Harmonic Mean Sample Size = 3.000.

**Biomass**

Duncan

| Carbon source | N | Subset for alpha = 0.05 | | | |
| --- | --- | --- | --- | --- | --- |
|  |  | 1 | 2 | 3 | 4 |
| Maltose | 3 | 3.68267 |  |  |  |
| Fructose | 3 |  | 4.65667 |  |  |
| Glucose | 3 |  |  | 6.18133 |  |
| Sucrose | 3 |  |  |  | 9.28067 |
| Sig. |  | 1.000 | 1.000 | 1.000 | 1.000 |

Means for groups in homogeneous subsets are displayed.

a Uses Harmonic Mean Sample Size = 3.000.

The effect of glucose concentration on astaxanthin content, astaxanthin yield and biomass of *R. paludigena* TL35-5

**ANOVA**

|  |  | Sum of Squares | df | Mean Square | F | Sig. |
| --- | --- | --- | --- | --- | --- | --- |
| Astaxanthin content | Between Groups | .084 | 4 | .021 | 585.162 | .000 |
|  | Within Groups | .000 | 10 | .000 |  |  |
|  | Total | .085 | 14 |  |  |  |
| Astaxanthin yield | Between Groups | 3.633 | 4 | .908 | 438.459 | .000 |
|  | Within Groups | .021 | 10 | .002 |  |  |
|  | Total | 3.654 | 14 |  |  |  |
| Biomass | Between Groups | 17.752 | 4 | 4.438 | 449789.500 | .000 |
|  | Within Groups | .000 | 10 | .000 |  |  |
|  | Total | 17.752 | 14 |  |  |  |

**Astaxanthin content**

Duncan

| Glucose concentration | N | Subset for alpha = 0.05 | | | | |
| --- | --- | --- | --- | --- | --- | --- |
|  |  | 1 | 2 | 3 | 4 | 5 |
| 50 g/L | 3 | .08467 |  |  |  |  |
| 40 g/L | 3 |  | .11567 |  |  |  |
| 30 g/L | 3 |  |  | .13733 |  |  |
| 20 g/L | 3 |  |  |  | .23500 |  |
| 10 g/L | 3 |  |  |  |  | .28233 |
| Sig. |  | 1.000 | 1.000 | 1.000 | 1.000 | 1.000 |

Means for groups in homogeneous subsets are displayed.

a Uses Harmonic Mean Sample Size = 3.000.

**Astaxanthin yield**

Duncan

| Glucose concentration | N | Subset for alpha = 0.05 | | | | |
| --- | --- | --- | --- | --- | --- | --- |
|  |  | 1 | 2 | 3 | 4 | 5 |
| 50 g/L | 3 | .73467 |  |  |  |  |
| 40 g/L | 3 |  | 1.18867 |  |  |  |
| 30 g/L | 3 |  |  | 1.35867 |  |  |
| 20 g/L | 3 |  |  |  | 1.81800 |  |
| 10 g/L | 3 |  |  |  |  | 2.14800 |
| Sig. |  | 1.000 | 1.000 | 1.000 | 1.000 | 1.000 |

Means for groups in homogeneous subsets are displayed.

a Uses Harmonic Mean Sample Size = 3.000.

**Biomass**

Duncan

| Glucose concentration | N | Subset for alpha = 0.05 | | | | |
| --- | --- | --- | --- | --- | --- | --- |
|  |  | 1 | 2 | 3 | 4 | 5 |
| 10 g/L | 3 | 7.61467 |  |  |  |  |
| 20 g/L | 3 |  | 7.74067 |  |  |  |
| 50 g/L | 3 |  |  | 8.65267 |  |  |
| 30 g/L | 3 |  |  |  | 9.88267 |  |
| 40 g/L | 3 |  |  |  |  | 10.28400 |
| Sig. |  | 1.000 | 1.000 | 1.000 | 1.000 | 1.000 |

Means for groups in homogeneous subsets are displayed.

a Uses Harmonic Mean Sample Size = 3.000.

The effect of nitrogen supplementation on astaxanthin content, astaxanthin yield and biomass of *R. paludigena* TL35-5

**ANOVA**

|  |  | Sum of Squares | df | Mean Square | F | Sig. |
| --- | --- | --- | --- | --- | --- | --- |
| Astaxanthin content | Between Groups | .034 | 3 | .011 | 218.429 | .000 |
|  | Within Groups | .000 | 8 | .000 |  |  |
|  | Total | .034 | 11 |  |  |  |
| Astaxanthin yield | Between Groups | 4.517 | 3 | 1.506 | 972.058 | .000 |
|  | Within Groups | .012 | 8 | .002 |  |  |
|  | Total | 4.529 | 11 |  |  |  |
| Biomass | Between Groups | 15.530 | 3 | 5.177 | 91352.916 | .000 |
|  | Within Groups | .000 | 8 | .000 |  |  |
|  | Total | 15.530 | 11 |  |  |  |

**Astaxanthin content**

Duncan

| Nitrogen source | N | Subset for alpha = 0.05 | | | |
| --- | --- | --- | --- | --- | --- |
|  |  | 1 | 2 | 3 | 4 |
| Urea | 3 | .27133 |  |  |  |
| Ammonium sulfate | 3 |  | .33700 |  |  |
| Control | 3 |  |  | .35733 |  |
| Ammonium nitrate | 3 |  |  |  | .42067 |
| Sig. |  | 1.000 | 1.000 | 1.000 | 1.000 |

Means for groups in homogeneous subsets are displayed.

a Uses Harmonic Mean Sample Size = 3.000.

**Astaxanthin yield**

Duncan

| Nitrogen source | N | Subset for alpha = 0.05 | | | |
| --- | --- | --- | --- | --- | --- |
|  |  | 1 | 2 | 3 | 4 |
| Urea | 3 | .91567 |  |  |  |
| Ammonium sulfate | 3 |  | 1.83767 |  |  |
| Control | 3 |  |  | 2.21800 |  |
| Ammonium nitrate | 3 |  |  |  | 2.55800 |
| Sig. |  | 1.000 | 1.000 | 1.000 | 1.000 |

Means for groups in homogeneous subsets are displayed.

a Uses Harmonic Mean Sample Size = 3.000.

**Biomass**

Duncan

| Nitrogen source | N | Subset for alpha = 0.05 | | | |
| --- | --- | --- | --- | --- | --- |
|  |  | 1 | 2 | 3 | 4 |
| Urea | 3 | 3.37333 |  |  |  |
| Ammonium sulfate | 3 |  | 5.45000 |  |  |
| Ammonium nitrate | 3 |  |  | 6.08467 |  |
| Control | 3 |  |  |  | 6.21000 |
| Sig. |  | 1.000 | 1.000 | 1.000 | 1.000 |

Means for groups in homogeneous subsets are displayed.

a Uses Harmonic Mean Sample Size = 3.000.

The effect of incubating temperature on astaxanthin content, astaxanthin yield and biomass of *R. paludigena* TL35-5

**ANOVA**

|  |  | Sum of Squares | df | Mean Square | F | Sig. |
| --- | --- | --- | --- | --- | --- | --- |
| Astaxanthin content | Between Groups | .025 | 3 | .008 | 343.577 | .000 |
|  | Within Groups | .000 | 8 | .000 |  |  |
|  | Total | .025 | 11 |  |  |  |
| Astaxanthin yield | Between Groups | .327 | 3 | .109 | 146.825 | .000 |
|  | Within Groups | .006 | 8 | .001 |  |  |
|  | Total | .333 | 11 |  |  |  |
| Biomass | Between Groups | 3.179 | 3 | 1.060 | 529863.278 | .000 |
|  | Within Groups | .000 | 8 | .000 |  |  |
|  | Total | 3.179 | 11 |  |  |  |

**Astaxanthin content**

Duncan

| Temperature | N | Subset for alpha = 0.05 | | |
| --- | --- | --- | --- | --- |
|  |  | 1 | 2 | 3 |
| 15 °C | 3 | .33667 |  |  |
| 20 °C | 3 |  | .40400 |  |
| 30 °C | 3 |  | .40533 |  |
| 25 °C | 3 |  |  | .46633 |
| Sig. |  | 1.000 | .750 | 1.000 |

Means for groups in homogeneous subsets are displayed.

a Uses Harmonic Mean Sample Size = 3.000.

**Astaxanthin yield**

Duncan

| Temperature | N | Subset for alpha = 0.05 | | |
| --- | --- | --- | --- | --- |
|  |  | 1 | 2 | 3 |
| 15 °C | 3 | 1.99267 |  |  |
| 30 °C | 3 | 2.00267 |  |  |
| 25 °C | 3 |  | 2.24800 |  |
| 20 °C | 3 |  |  | 2.38067 |
| Sig. |  | .665 | 1.000 | 1.000 |

Means for groups in homogeneous subsets are displayed.

a Uses Harmonic Mean Sample Size = 3.000.

**Biomass**

Duncan

| Temperature | N | Subset for alpha = 0.05 | | | |
| --- | --- | --- | --- | --- | --- |
|  |  | 1 | 2 | 3 | 4 |
| 25 °C | 3 | 4.82067 |  |  |  |
| 30 °C | 3 |  | 4.94200 |  |  |
| 20 °C | 3 |  |  | 5.89333 |  |
| 15 °C | 3 |  |  |  | 5.92067 |
| Sig. |  | 1.000 | 1.000 | 1.000 | 1.000 |

Means for groups in homogeneous subsets are displayed.

a Uses Harmonic Mean Sample Size = 3.000.

The effect of initial pH on astaxanthin content, astaxanthin yield and biomass of *R. paludigena* TL35-5

**ANOVA**

|  |  | Sum of Squares | df | Mean Square | F | Sig. |
| --- | --- | --- | --- | --- | --- | --- |
| Astaxanthin content | Between Groups | .003 | 4 | .001 | 15.200 | .000 |
|  | Within Groups | .000 | 10 | .000 |  |  |
|  | Total | .003 | 14 |  |  |  |
| Astaxanthin yield | Between Groups | .309 | 4 | .077 | 89.231 | .000 |
|  | Within Groups | .009 | 10 | .001 |  |  |
|  | Total | .318 | 14 |  |  |  |
| Biomass | Between Groups | .803 | 4 | .201 | 83606.667 | .000 |
|  | Within Groups | .000 | 10 | .000 |  |  |
|  | Total | .803 | 14 |  |  |  |

**Astaxanthin content**

Duncan

| pH | N | Subset for alpha = 0.05 | | |
| --- | --- | --- | --- | --- |
|  |  | 1 | 2 | 3 |
| pH 8.5 | 3 | .46800 |  |  |
| pH 4.5 | 3 |  | .48300 |  |
| pH 7.5 | 3 |  |  | .49667 |
| pH 5.5 | 3 |  |  | .50200 |
| pH 6.5 | 3 |  |  | .50300 |
| Sig. |  | 1.000 | 1.000 | .290 |

Means for groups in homogeneous subsets are displayed.

a Uses Harmonic Mean Sample Size = 3.000.

**Astaxanthin yield**

Duncan

| pH | N | Subset for alpha = 0.05 | | | |
| --- | --- | --- | --- | --- | --- |
|  |  | 1 | 2 | 3 | 4 |
| pH 4.5 | 3 | 2.03067 |  |  |  |
| pH 8.5 | 3 |  | 2.12300 |  |  |
| pH 7.5 | 3 |  |  | 2.18000 |  |
| pH 5.5 | 3 |  |  | 2.20033 |  |
| pH 6.5 | 3 |  |  |  | 2.46100 |
| Sig. |  | 1.000 | 1.000 | .417 | 1.000 |

Means for groups in homogeneous subsets are displayed.

a Uses Harmonic Mean Sample Size = 3.000.

**Biomass**

Duncan

| pH | N | Subset for alpha = 0.05 | | | | |
| --- | --- | --- | --- | --- | --- | --- |
|  |  | 1 | 2 | 3 | 4 | 5 |
| pH 4.5 | 3 | 4.20333 |  |  |  |  |
| pH 5.5 | 3 |  | 4.38200 |  |  |  |
| pH 7.5 | 3 |  |  | 4.38733 |  |  |
| pH 8.5 | 3 |  |  |  | 4.53533 |  |
| pH 6.5 | 3 |  |  |  |  | 4.89200 |
| Sig. |  | 1.000 | 1.000 | 1.000 | 1.000 | 1.000 |

Means for groups in homogeneous subsets are displayed.

a Uses Harmonic Mean Sample Size = 3.000.

The effect of incubation time on astaxanthin content, astaxanthin yield and biomass of *R. paludigena* TL35-5

**ANOVA**

|  |  | Sum of Squares | df | Mean Square | F | Sig. |
| --- | --- | --- | --- | --- | --- | --- |
| Astaxanthin content | Between Groups | .309 | 3 | .103 | 5725.461 | .000 |
|  | Within Groups | .000 | 8 | .000 |  |  |
|  | Total | .309 | 11 |  |  |  |
| Astaxanthin yield | Between Groups | 18.399 | 3 | 6.133 | 15906.137 | .000 |
|  | Within Groups | .003 | 8 | .000 |  |  |
|  | Total | 18.403 | 11 |  |  |  |
| Biomass | Between Groups | 31.826 | 3 | 10.609 | 1326068.111 | .000 |
|  | Within Groups | .000 | 8 | .000 |  |  |
|  | Total | 31.826 | 11 |  |  |  |
| Astaxanthin productivity | Between Groups | .778 | 3 | .259 | 2172.481 | .000 |
|  | Within Groups | .001 | 8 | .000 |  |  |
|  | Total | .779 | 11 |  |  |  |

**Astaxanthin content**

Duncan

| Time | N | Subset for alpha = 0.05 | | | |
| --- | --- | --- | --- | --- | --- |
|  |  | 1 | 2 | 3 | 4 |
| 1 day | 3 | .09333 |  |  |  |
| 3 days | 3 |  | .37767 |  |  |
| 7 days | 3 |  |  | .40067 |  |
| 5 days | 3 |  |  |  | .53400 |
| Sig. |  | 1.000 | 1.000 | 1.000 | 1.000 |

Means for groups in homogeneous subsets are displayed.

a Uses Harmonic Mean Sample Size = 3.000.

**Astaxanthin yield**

Duncan

| Time | N | Subset for alpha = 0.05 | | | |
| --- | --- | --- | --- | --- | --- |
|  |  | 1 | 2 | 3 | 4 |
| 1 day | 3 | .27867 |  |  |  |
| 7 days | 3 |  | 2.40833 |  |  |
| 3 days | 3 |  |  | 2.63767 |  |
| 5 days | 3 |  |  |  | 3.68933 |
| Sig. |  | 1.000 | 1.000 | 1.000 | 1.000 |

Means for groups in homogeneous subsets are displayed.

a Uses Harmonic Mean Sample Size = 3.000.

**Biomass**

Duncan

| Time | N | Subset for alpha = 0.05 | | | |
| --- | --- | --- | --- | --- | --- |
|  |  | 1 | 2 | 3 | 4 |
| 1 day | 3 | 2.97533 |  |  |  |
| 7 days | 3 |  | 6.00867 |  |  |
| 5 days | 3 |  |  | 6.90933 |  |
| 3 days | 3 |  |  |  | 6.97600 |
| Sig. |  | 1.000 | 1.000 | 1.000 | 1.000 |

Means for groups in homogeneous subsets are displayed.

a Uses Harmonic Mean Sample Size = 3.000.

**Astaxanthin productivity**

Duncan

| Time | N | Subset for alpha = 0.05 | | | |
| --- | --- | --- | --- | --- | --- |
|  |  | 1 | 2 | 3 | 4 |
| 1 day | 3 | .27867 |  |  |  |
| 7 days | 3 |  | .34400 |  |  |
| 5 days | 3 |  |  | .73800 |  |
| 3 days | 3 |  |  |  | .87900 |
| Sig. |  | 1.000 | 1.000 | 1.000 | 1.000 |

Means for groups in homogeneous subsets are displayed.

a Uses Harmonic Mean Sample Size = 3.000.
