# Supplementary material for: Comparative genomic analysis and optimization of astaxanthin production of Rhodotorula paludigena TL35-5 and Rhodotorula sampaioana PL61-2
Source: PLoS One. 2024 Jul 12;19(7):e0304699. doi: 10.1371/journal.pone.0304699 (PMC11244826; doi:10.1371/journal.pone.0304699)
Supplement: S2 File — (DOCX) [file pone.0304699.s005.docx]

The effect of different carbon sources on astaxanthin content, astaxanthin yield and biomass of *R. sampaioana* PL61-2

**ANOVA**

|  |  | Sum of Squares | df | Mean Square | F | Sig. |
| --- | --- | --- | --- | --- | --- | --- |
| Astaxanthin content | Between Groups | .044 | 3 | .015 | 427.165 | .000 |
|  | Within Groups | .000 | 8 | .000 |  |  |
|  | Total | .044 | 11 |  |  |  |
| Astaxanthin yield | Between Groups | .181 | 3 | .060 | 164.452 | .000 |
|  | Within Groups | .003 | 8 | .000 |  |  |
|  | Total | .183 | 11 |  |  |  |
| Biomass | Between Groups | 15.259 | 3 | 5.086 | 586877.064 | .000 |
|  | Within Groups | .000 | 8 | .000 |  |  |
|  | Total | 15.259 | 11 |  |  |  |

**Astaxanthin content**

Duncan

| Carbon source | N | Subset for alpha = 0.05 | | | |
| --- | --- | --- | --- | --- | --- |
|  |  | 1 | 2 | 3 | 4 |
| Maltose | 3 | .03933 |  |  |  |
| Sucrose | 3 |  | .07567 |  |  |
| Fructose | 3 |  |  | .12233 |  |
| Glucose | 3 |  |  |  | .20133 |
| Sig. |  | 1.000 | 1.000 | 1.000 | 1.000 |

Means for groups in homogeneous subsets are displayed.

a Uses Harmonic Mean Sample Size = 3.000.

**Astaxanthin yield**

Duncan

| Carbon source | N | Subset for alpha = 0.05 | |
| --- | --- | --- | --- |
|  |  | 1 | 2 |
| Maltose | 3 | .20667 |  |
| Sucrose | 3 | .21300 |  |
| Fructose | 3 |  | .44633 |
| Glucose | 3 |  | .46333 |
| Sig. |  | .696 | .308 |

Means for groups in homogeneous subsets are displayed.

a Uses Harmonic Mean Sample Size = 3.000.

**Biomass**

Duncan

| Carbon source | N | Subset for alpha = 0.05 | | | |
| --- | --- | --- | --- | --- | --- |
|  |  | 1 | 2 | 3 | 4 |
| Glucose | 3 | 2.30600 |  |  |  |
| Sucrose | 3 |  | 2.82200 |  |  |
| Fructose | 3 |  |  | 3.64667 |  |
| Maltose | 3 |  |  |  | 5.28333 |
| Sig. |  | 1.000 | 1.000 | 1.000 | 1.000 |

Means for groups in homogeneous subsets are displayed.

a Uses Harmonic Mean Sample Size = 3.000.

a Uses Harmonic Mean Sample Size = 3.000.

The effect of glucose concentration on astaxanthin content, astaxanthin yield and biomass of *R. sampaioana* PL61-2

**ANOVA**

|  |  | Sum of Squares | df | Mean Square | F | Sig. |
| --- | --- | --- | --- | --- | --- | --- |
| Astaxanthin content | Between Groups | .008 | 4 | .002 | 798.936 | .000 |
|  | Within Groups | .000 | 10 | .000 |  |  |
|  | Total | .008 | 14 |  |  |  |
| Astaxanthin yield | Between Groups | 3.874 | 4 | .968 | 221.650 | .000 |
|  | Within Groups | .044 | 10 | .004 |  |  |
|  | Total | 3.918 | 14 |  |  |  |
| Biomass | Between Groups | 78.580 | 4 | 19.645 | 74.179 | .000 |
|  | Within Groups | 2.648 | 10 | .265 |  |  |
|  | Total | 81.229 | 14 |  |  |  |

**Astaxanthin content**

Duncan

| Glucose concentration | N | Subset for alpha = 0.05 | | | | |
| --- | --- | --- | --- | --- | --- | --- |
|  |  | 1 | 2 | 3 | 4 | 5 |
| 50 g/L | 3 | .14400 |  |  |  |  |
| 10 g/L | 3 |  | .15033 |  |  |  |
| 20 g/L | 3 |  |  | .18100 |  |  |
| 30 g/L | 3 |  |  |  | .18867 |  |
| 40 g/L | 3 |  |  |  |  | .20633 |
| Sig. |  | 1.000 | 1.000 | 1.000 | 1.000 | 1.000 |

Means for groups in homogeneous subsets are displayed.

a Uses Harmonic Mean Sample Size = 3.000.

**Astaxanthin yield**

Duncan

| Glucose concentration | N | Subset for alpha = 0.05 | | | | |
| --- | --- | --- | --- | --- | --- | --- |
|  |  | 1 | 2 | 3 | 4 | 5 |
| 10 g/L | 3 | .54533 |  |  |  |  |
| 50 g/L | 3 |  | 1.30300 |  |  |  |
| 20 g/L | 3 |  |  | 1.46000 |  |  |
| 30 g/L | 3 |  |  |  | 1.85633 |  |
| 40 g/L | 3 |  |  |  |  | 1.98667 |
| Sig. |  | 1.000 | 1.000 | 1.000 | 1.000 | 1.000 |

Means for groups in homogeneous subsets are displayed.

a Uses Harmonic Mean Sample Size = 3.000.

**Biomass**

Duncan

| Glucose concentration | N | Subset for alpha = 0.05 | | |
| --- | --- | --- | --- | --- |
|  |  | 1 | 2 | 3 |
| 10 g/L | 3 | 3.62800 |  |  |
| 20 g/L | 3 |  | 8.07133 |  |
| 50 g/L | 3 |  |  | 9.05867 |
| 40 g/L | 3 |  |  | 9.61333 |
| 30 g/L | 3 |  |  | 9.83267 |
| Sig. |  | 1.000 | 1.000 | .109 |

Means for groups in homogeneous subsets are displayed.

a Uses Harmonic Mean Sample Size = 3.000.

The effect of nitrogen supplementation on astaxanthin content, astaxanthin yield and biomass of *R. sampaioana*

PL61-2

**ANOVA**

|  |  | Sum of Squares | df | Mean Square | F | Sig. |
| --- | --- | --- | --- | --- | --- | --- |
| Astaxanthin content | Between Groups | .007 | 3 | .002 | 784.459 | .000 |
|  | Within Groups | .000 | 8 | .000 |  |  |
|  | Total | .007 | 11 |  |  |  |
| Astaxanthin yield | Between Groups | .807 | 3 | .269 | 825.981 | .000 |
|  | Within Groups | .003 | 8 | .000 |  |  |
|  | Total | .810 | 11 |  |  |  |
| Biomass | Between Groups | .249 | 3 | .083 | 718.700 | .000 |
|  | Within Groups | .001 | 8 | .000 |  |  |
|  | Total | .250 | 11 |  |  |  |

**Astaxanthin content**

Duncan

| Nitrogen source | N | Subset for alpha = 0.05 | | | |
| --- | --- | --- | --- | --- | --- |
|  |  | 1 | 2 | 3 | 4 |
| Control | 3 | .17067 |  |  |  |
| Ammonium nitrate | 3 |  | .19467 |  |  |
| Ammonium sulfate | 3 |  |  | .21333 |  |
| Urea | 3 |  |  |  | .23767 |
| Sig. |  | 1.000 | 1.000 | 1.000 | 1.000 |

Means for groups in homogeneous subsets are displayed.

a Uses Harmonic Mean Sample Size = 3.000.

**Astaxanthin yield**

Duncan

| Nitrogen source | N | Subset for alpha = 0.05 | | | |
| --- | --- | --- | --- | --- | --- |
|  |  | 1 | 2 | 3 | 4 |
| Control | 3 | 1.64533 |  |  |  |
| Ammonium nitrate | 3 |  | 1.92000 |  |  |
| Ammonium sulfate | 3 |  |  | 2.05233 |  |
| Urea | 3 |  |  |  | 2.36633 |
| Sig. |  | 1.000 | 1.000 | 1.000 | 1.000 |

Means for groups in homogeneous subsets are displayed.

a Uses Harmonic Mean Sample Size = 3.000.

**Biomass**

Duncan

| Nitrogen source | N | Subset for alpha = 0.05 | | | |
| --- | --- | --- | --- | --- | --- |
|  |  | 1 | 2 | 3 | 4 |
| Ammonium sulfate | 3 | 9.62267 |  |  |  |
| Control | 3 |  | 9.64533 |  |  |
| Ammonium nitrate | 3 |  |  | 9.85667 |  |
| Urea | 3 |  |  |  | 9.96533 |
| Sig. |  | 1.000 | 1.000 | 1.000 | 1.000 |

Means for groups in homogeneous subsets are displayed.

a Uses Harmonic Mean Sample Size = 3.000.

The effect of incubating temperature on astaxanthin content, astaxanthin yield and biomass of *R. sampaioana*

PL61-2

**ANOVA**

|  |  | Sum of Squares | df | Mean Square | F | Sig. |
| --- | --- | --- | --- | --- | --- | --- |
| Astaxanthin content | Between Groups | .022 | 3 | .007 | 35.918 | .000 |
|  | Within Groups | .002 | 8 | .000 |  |  |
|  | Total | .023 | 11 |  |  |  |
| Astaxanthin yield | Between Groups | 7.090 | 3 | 2.363 | 104.173 | .000 |
|  | Within Groups | .181 | 8 | .023 |  |  |
|  | Total | 7.272 | 11 |  |  |  |
| Biomass | Between Groups | 28.359 | 3 | 9.453 | 3544838.333 | .000 |
|  | Within Groups | .000 | 8 | .000 |  |  |
|  | Total | 28.359 | 11 |  |  |  |

**Astaxanthin content**

Duncan

| Temperature | N | Subset for alpha = 0.05 | | |
| --- | --- | --- | --- | --- |
|  |  | 1 | 2 | 3 |
| 30 °C | 3 | .19067 |  |  |
| 15 °C | 3 |  | .24900 |  |
| 25 °C | 3 |  | .26200 |  |
| 20 °C | 3 |  |  | .31000 |
| Sig. |  | 1.000 | .294 | 1.000 |

Means for groups in homogeneous subsets are displayed.

a Uses Harmonic Mean Sample Size = 3.000.

**Astaxanthin yield**

Duncan

| Temperature | N | Subset for alpha = 0.05 | | | |
| --- | --- | --- | --- | --- | --- |
|  |  | 1 | 2 | 3 | 4 |
| 30 °C | 3 | 1.48867 |  |  |  |
| 15 °C | 3 |  | 1.98067 |  |  |
| 25 °C | 3 |  |  | 2.72300 |  |
| 20 °C | 3 |  |  |  | 3.52067 |
| Sig. |  | 1.000 | 1.000 | 1.000 | 1.000 |

Means for groups in homogeneous subsets are displayed.

a Uses Harmonic Mean Sample Size = 3.000.

**Biomass**

Duncan

| Temperature | N | Subset for alpha = 0.05 | | | |
| --- | --- | --- | --- | --- | --- |
|  |  | 1 | 2 | 3 | 4 |
| 30 °C | 3 | 7.80200 |  |  |  |
| 15 °C | 3 |  | 7.95267 |  |  |
| 25 °C | 3 |  |  | 10.38000 |  |
| 20 °C | 3 |  |  |  | 11.36133 |
| Sig. |  | 1.000 | 1.000 | 1.000 | 1.000 |

Means for groups in homogeneous subsets are displayed.

a Uses Harmonic Mean Sample Size = 3.000.

The effect of different carbon sources on astaxanthin content, astaxanthin yield and biomass of *R. sampaioana*

PL61-2

**ANOVA**

|  |  | Sum of Squares | df | Mean Square | F | Sig. |
| --- | --- | --- | --- | --- | --- | --- |
| Astaxanthin content | Between Groups | .019 | 4 | .005 | 187.636 | .000 |
|  | Within Groups | .000 | 10 | .000 |  |  |
|  | Total | .019 | 14 |  |  |  |
| Astaxanthin yield | Between Groups | 2.558 | 4 | .640 | 250.988 | .000 |
|  | Within Groups | .025 | 10 | .003 |  |  |
|  | Total | 2.584 | 14 |  |  |  |
| Biomass | Between Groups | 5.297 | 4 | 1.324 | 198642.760 | .000 |
|  | Within Groups | .000 | 10 | .000 |  |  |
|  | Total | 5.297 | 14 |  |  |  |

**Astaxanthin content**

Duncan

| pH | N | Subset for alpha = 0.05 | | |
| --- | --- | --- | --- | --- |
|  |  | 1 | 2 | 3 |
| pH 6.5 | 3 | .24900 |  |  |
| pH 4.5 | 3 | .25233 |  |  |
| pH 8.5 | 3 | .25633 |  |  |
| pH 5.5 | 3 |  | .27267 |  |
| pH 7.5 | 3 |  |  | .34400 |
| Sig. |  | .118 | 1.000 | 1.000 |

Means for groups in homogeneous subsets are displayed.

a Uses Harmonic Mean Sample Size = 3.000.

**Astaxanthin yield**

Duncan

| pH | N | Subset for alpha = 0.05 | | | |
| --- | --- | --- | --- | --- | --- |
|  |  | 1 | 2 | 3 | 4 |
| pH 4.5 | 3 | 2.24100 |  |  |  |
| pH 6.5 | 3 |  | 2.50100 |  |  |
| pH 5.5 | 3 |  |  | 2.69167 |  |
| pH 8.5 | 3 |  |  | 2.74433 |  |
| pH 7.5 | 3 |  |  |  | 3.47800 |
| Sig. |  | 1.000 | 1.000 | .230 | 1.000 |

Means for groups in homogeneous subsets are displayed.

a Uses Harmonic Mean Sample Size = 3.000.

**Biomass**

Duncan

| pH | N | Subset for alpha = 0.05 | | | | |
| --- | --- | --- | --- | --- | --- | --- |
|  |  | 1 | 2 | 3 | 4 | 5 |
| pH 4.5 | 3 | 8.88400 |  |  |  |  |
| pH 5.5 | 3 |  | 9.87600 |  |  |  |
| pH 6.5 | 3 |  |  | 10.04400 |  |  |
| pH 7.5 | 3 |  |  |  | 10.12467 |  |
| pH 8.5 | 3 |  |  |  |  | 10.71600 |
| Sig. |  | 1.000 | 1.000 | 1.000 | 1.000 | 1.000 |

Means for groups in homogeneous subsets are displayed.

a Uses Harmonic Mean Sample Size = 3.000.

The effect of incubation time sources on astaxanthin content, astaxanthin yield and biomass of *R. sampaioana*

PL61-2

**ANOVA**

|  |  | Sum of Squares | df | Mean Square | F | Sig. |
| --- | --- | --- | --- | --- | --- | --- |
| Astaxanthin content | Between Groups | .063 | 3 | .021 | 328.666 | .000 |
|  | Within Groups | .001 | 8 | .000 |  |  |
|  | Total | .063 | 11 |  |  |  |
| Astaxanthin yield | Between Groups | 29.107 | 3 | 9.702 | 744.996 | .000 |
|  | Within Groups | .104 | 8 | .013 |  |  |
|  | Total | 29.211 | 11 |  |  |  |
| Biomass | Between Groups | 139.181 | 3 | 46.394 | 17397643.792 | .000 |
|  | Within Groups | .000 | 8 | .000 |  |  |
|  | Total | 139.181 | 11 |  |  |  |
| Astaxanthin productivity | Between Groups | 3.496 | 3 | 1.165 | 785.155 | .000 |
|  | Within Groups | .012 | 8 | .001 |  |  |
|  | Total | 3.508 | 11 |  |  |  |

**Astaxanthin content**

Duncan

| Time | N | Subset for alpha = 0.05 | | | |
| --- | --- | --- | --- | --- | --- |
|  |  | 1 | 2 | 3 | 4 |
| 1 day | 3 | .16667 |  |  |  |
| 7 days | 3 |  | .21967 |  |  |
| 3 days | 3 |  |  | .29800 |  |
| 5 days | 3 |  |  |  | .35533 |
| Sig. |  | 1.000 | 1.000 | 1.000 | 1.000 |

Means for groups in homogeneous subsets are displayed.

a Uses Harmonic Mean Sample Size = 3.000.

**Astaxanthin yield**

Duncan

| Time | N | Subset for alpha = 0.05 | | | |
| --- | --- | --- | --- | --- | --- |
|  |  | 1 | 2 | 3 | 4 |
| 7 days | 3 | 1.31467 |  |  |  |
| 1 day | 3 |  | 1.54100 |  |  |
| 3 days | 3 |  |  | 4.38333 |  |
| 5 days | 3 |  |  |  | 4.67967 |
| Sig. |  | 1.000 | 1.000 | 1.000 | 1.000 |

Means for groups in homogeneous subsets are displayed.

a Uses Harmonic Mean Sample Size = 3.000.

**Biomass**

Duncan

| Time | N | Subset for alpha = 0.05 | | | |
| --- | --- | --- | --- | --- | --- |
|  |  | 1 | 2 | 3 | 4 |
| 1 day | 3 | 9.2480 |  |  |  |
| 7 days | 3 |  | 14.7027 |  |  |
| 5 days | 3 |  |  | 13.1680 |  |
| 3 days | 3 |  |  |  | 5.9887 |
| Sig. |  | 1.000 | 1.000 | 1.000 | 1.000 |

Means for groups in homogeneous subsets are displayed.

a Uses Harmonic Mean Sample Size = 3.000.

**Astaxanthin productivity**

Duncan

| Time | N | Subset for alpha = 0.05 | | | |
| --- | --- | --- | --- | --- | --- |
|  |  | 1 | 2 | 3 | 4 |
| 7 days | 3 | .18767 |  |  |  |
| 5 days | 3 |  | .93600 |  |  |
| 3 days | 3 |  |  | 1.46100 |  |
| 1 day | 3 |  |  |  | 1.54100 |
| Sig. |  | 1.000 | 1.000 | 1.000 | 1.000 |

Means for groups in homogeneous subsets are displayed.

a Uses Harmonic Mean Sample Size = 3.000.
